# Understanding the Interaction of Gluconamides and Gluconates with Amino Acids in Hair Care

**DOI:** 10.1021/acs.cgd.2c00753

**Published:** 2022-09-20

**Authors:** Luke I. Chambers, Dmitry S. Yufit, Osama M. Musa, Jonathan W. Steed

**Affiliations:** † Department of Chemistry, Lower Mountjoy, Durham University, Stockton Road, Durham DH1 3LE, U.K.; ‡ Ashland LLC, 1005 Route 202/206, Bridgewater, New Jersey 08807, United States

## Abstract

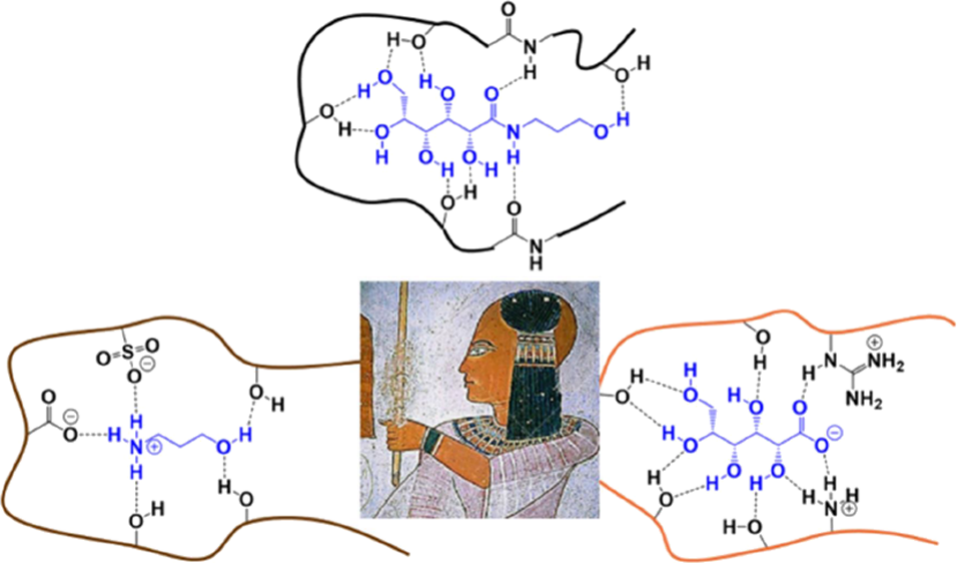

A hair care mixture formed from a gluconamide derivative and 3-hydroxypropyl ammonium gluconate is known to strengthen hair fibers; however, the mechanism by which the mixture affects hair is unknown. To give insight into the aggregation of the target gluconamide and potential interactions between the gluconate-derived mixture and hair fibers, a range of systems were characterized by X-ray crystallography namely two polymorphic forms of the target gluconamide and three salts of 3-hydroxypropylammonium with sulfuric acid, methane sulfonic acid, and oxalic acid. The gluconamide proves to aggregate and becomes a supramolecular gelator in aniline and benzyl alcohol solution. The resulting gels were characterized by rheology, scanning electron microscopy, proton nuclear magnetic resonance, Fourier transform infrared spectroscopy, and powder X-ray diffraction.

## Abstract 2

This work examines the mechanism of hair fiber strengthening and gelation behavior of a gluconamide derivative by examining its aggregation and potential interactions between the gluconate-derived mixture and hair fiber.

## Introduction

A human hair fiber is around 50–100 μm in diameter, and it is composed of three main parts: cuticle, cortex, and medulla.^1,2^ The cuticle is the outer barrier protecting the cortex from external damage^1,3−7^ The cortex makes up most of the hair mass and is responsible for the high tensile strength^1,8−13^ while the inner part of the hair fiber is the medulla which provides a negligible contribution to its mechanical strength.^14,15^ Overall, the main chemical component by weight is protein composing 65–95% of the hair.^1^ The predominant proteins present are keratins which act as the structural building blocks of hair as well as other materials such as skin and nails.^16^ Human hair is formed from alpha keratins which are in an alpha-helix conformation and can be divided into two types, type I which is smaller (44–46 kDa) and more acidic and type II which is larger (50–60 kDa) and slightly basic or neutral.^1,17^ Keratin proteins can also be divided into type “a” or type “b”, with type “a” being hard keratins found in hair and type “b” being soft keratins found in the skin.^18^ Keratin proteins found in human hair contain more cysteine residues and fewer glycine residues compared to other keratins.^19^ The higher cysteine content causes increased disulfide bridge formation, resulting in greater mechanical strength, and thermal and chemical resistance.^20^ The strength of the structures formed from keratin is also related to the formation of hydrogen bonds, coulombic interactions, van der Waals forces, and hydrophobic interactions present between the different amino acid residues.^21^ These interactions can take place either between two separate chains or two portions of the same chain.

Hair can be damaged in a variety of ways including environmental, chemical, overwashing, or thermal damage.^20^ The amount and type of melanin pigments present determine the color of the hair. Oxidizing agents used in bleaching can oxidize and destroy the chromophore groups of melanin.^22^ The oxidizing agents also mechanically weaken hair by oxidizing the cysteine residues into cysteic acid which breaks the disulfide bridge which is usually formed between two cysteine residues.^23^Table 1 shows the changes in the amino acid composition between bleached and nonbleached hair. The two most significant changes are the drop in half cystine residues and the increase in cysteic acid residues.^20^

**Table 1:** Amino Acids in Bleached and Nonbleached Hair^20^

amino acid	micromoles per gram of hair	
	nonbleached hair	bleached hair
aspartic acid	437	432
threonine	616	588
serine	1085	973
glutamic acid	1030	999
proline	639	582
glycine	450	415
alanine	370	357
half cystine	1509	731
valine	487	464
methionine	50	38
isoleucine	227	220
leucine	509	485
tyrosine	183	146
phenylalanine	139	129
cysteic acid	27	655
lysine	198	180
histidine	65	55
arginine	511	486

**Figure 1:**
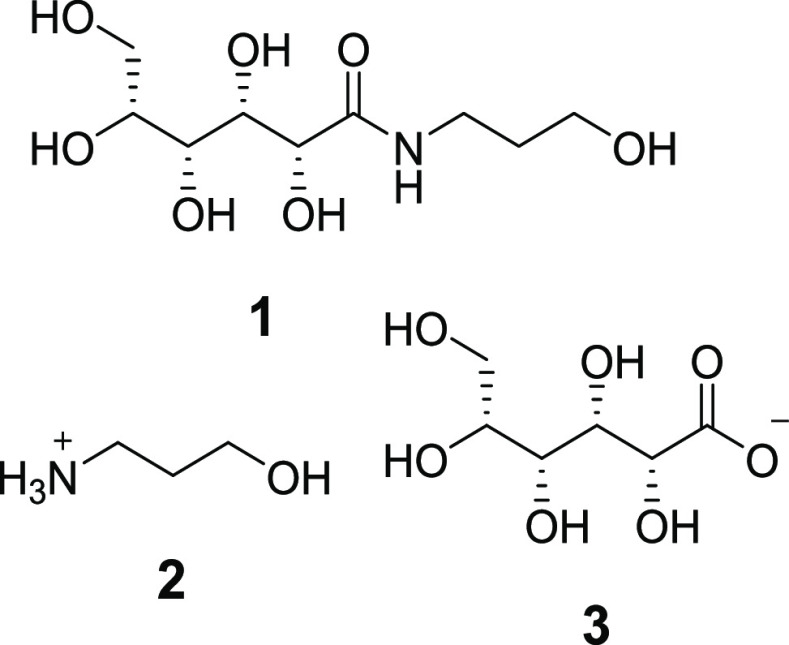
Components of the commercial fiberHance bm haircare solution: hydroxypropyl-l-gluconamide (**1**) and hydroxypropylammonium l-gluconate (**2** and **3**).

A hair treatment was reported in 2017 based on a range of gluconamides and their corresponding alkyl ammonium gluconate salts which were found to strengthen and repair damaged hair and prevent color leaching during drying.^24−26^ The compositions comprise l-gluconic acid (GLA) and a range of different amines including ethylenediamine, ethanolamine, 3-amino-1-propanol, and tris(hydroxymethyl)aminomethane).^24−26^ One of the compositions formed from 3-amino-1-propanol (3AP) and l-gluconic acid proved to provide the greatest strength to hair fibers^24−26^ and forms the basis of a commercial product comprising a 50 wt % aqueous solution called fiberHance bm solution,^27^ a mixture of hydroxypropyl-l-gluconamide (**1**), and hydroxypropylammonium l-gluconate (**2** and **3**) (Figure 1). The gluconamide **1** is initially present in a 1:1 molar ratio with the gluconate salt but converts into **2** and **3** in solution, particularly under basic conditions.

Sugars such as sucrose can stabilize the secondary structure of proteins, which may be related to the hair strengthening properties of **1**.^28^ Both the amide and the salt components are assumed to permeate the cuticle and reach the cortex.^29^ The exact nature of how this mixture acts to strengthen hair is currently unknown, but it is speculated that a range of intermolecular bonds including hydrogen bonds and ionic interactions are formed with amino acid residues in the keratin proteins.^29^ This work aims to examine the assembly mode of compound **1** and give some insight into potential interactions between **1**, **2**, and **3** and the amino acids present in hair. This aim has been addressed by examination of the single-crystal structures of **1** itself and a range of systems mimicking the substituent groups present in amino acid residues.

## Results and Discussion

### Gluconamide Structures

**Figure 2:**
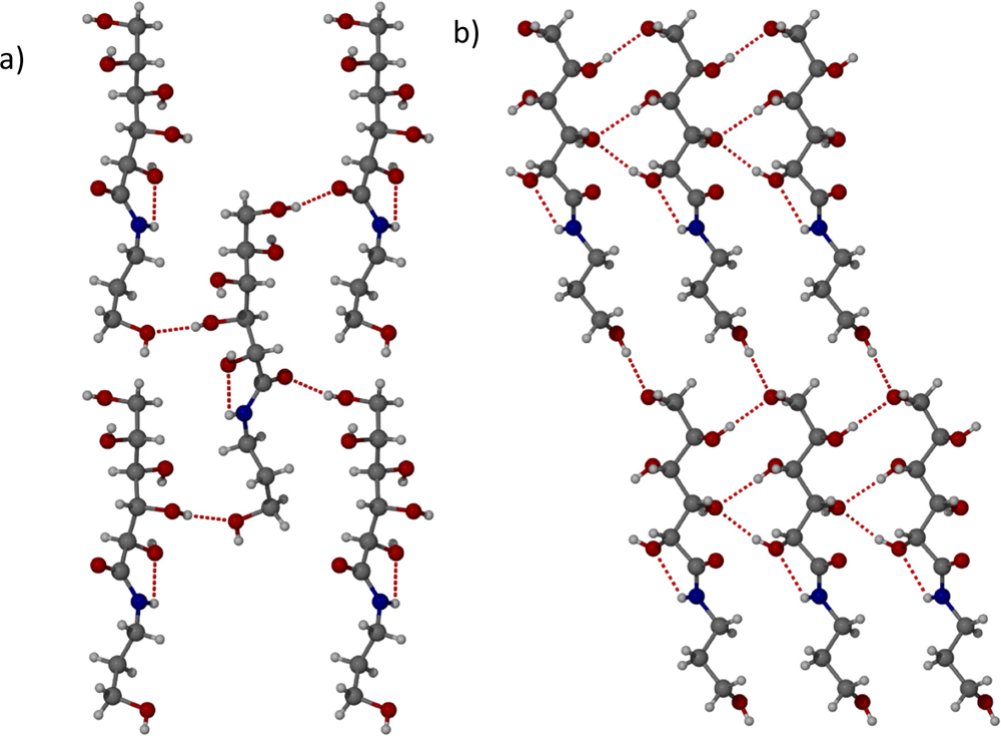
X-ray crystal structure of form I of compound **1** showing the hydrogen bonding in the (a) (100) and (b) (001) crystallographic planes.

Compound **1** was separated from the commercial aqueous haircare mixture by slow evaporation which resulted in crystals of one of two polymorphs (form I) suitable for single-crystal X-ray diffraction (SXRD). The X-ray crystal structure is in the Sohncke space group *P*2_1_ consistent with a single enantiomer of the gluconamide and contains one molecule of amide **1** in the asymmetric unit. The molecular structure of form I (Figure 2) involves a strained intramolecular hydrogen bond, forming a five-membered ring between the hydrogen atom from the amide group and the oxygen atom on the alcohol group next to the carbonyl group with an N···O distance of 2.5984(19) Å. The amide NH proton does not form any intermolecular hydrogen bonds. Form I does display extensive intermolecular hydrogen bonding from the OH groups with one molecule of **1** interacting with seven different neighbors. The alcohol groups form six different hydrogen bonds with other alcohol groups, and the range of O···O distances are 2.7583(18)–2.8448(18) Å. In addition, the carbonyl oxygen atom forms a strong hydrogen bond with an alcohol group on an adjacent molecule with an O···O distance of 2.6767(18) Å. An R_2_^1^(8) hydrogen bonding motif forms between two molecules of **1** which can be observed in the (001) crystallographic plane (Figure 2b). The opposite enantiomer of **1** was synthesized by mixing aqueous d-gluconic acid with 3AP in a 1:1 molar ratio and leaving the solution to evaporate. This process produced crystals which were analyzed by SXRD which revealed that the d-enantiomer forms an isomorphous crystal to form I under these conditions.

**Figure 3:**
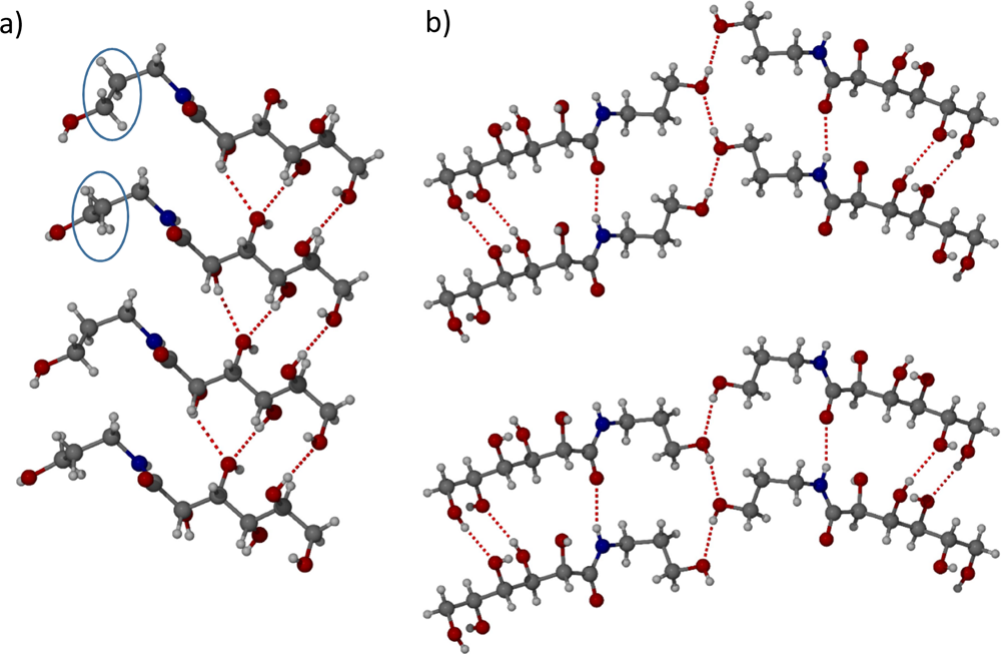
X-ray crystal structure of **1** form II. (a) Hydrogen bonded chain motif with the C1–C2 bond highlighted in a blue circle. (b) Two crystallographically independent molecules in the asymmetric unit form two separate chains along the terminal OH group.

A second polymorph of **1**, form II (Figure 3), was obtained by slow evaporation of an ethanol solution of **1** in the presence of aniline in a 1:5.5 molar ratio. Form II also adopts space group *P*2_1_, but the asymmetric unit contains two molecules of **1** in two different conformations (a conformational isomorph).^30^ The difference between the two molecules is in the torsion angle from the carbonyl to the C1–C2 bond (O2–C4–C2–C1) (Figure 3a) which is gauche in one (47.3°) and antigauche in the other (164.9 °). Both conformers differ from Form I which has a more extended conformation with the analogous torsion angle being 32.8°. Also unlike form I, the amide NH group takes part in an intermolecular interaction (Figure 3b) with the carbonyl group of an adjacent molecule with N···O distances of 2.840(4) and 2.834(4) Å for the two crystallographically independent molecules. The intramolecular NH···O contact is correspondingly slightly longer at 2.65 Å (average). The intermolecular amide hydrogen bond gives an infinite chain, similar to the β-sheet structure of proteins suggesting aggregation potential. The alcohol group from the 3AP group of **1** forms a repeating chain of hydrogen bonds with O···O distances of 2.790(2) and 2.787(2) Å, in which each crystallographically unique molecule is part of a separate chain (Figure 3b). The other hydrogen bonds take place between the other alcohol groups with O···O distances between 2.688(3) and 2.936(4) Å which are similar to form I. Each molecule of **1** is bonded to seven other molecules of **1** in the same way as form I. The same R_2_^1^(8) hydrogen bonding motifs observed in form I are also present in form II as shown in the (120) crystallographic plane (Figure 3a).

**Figure 4:**
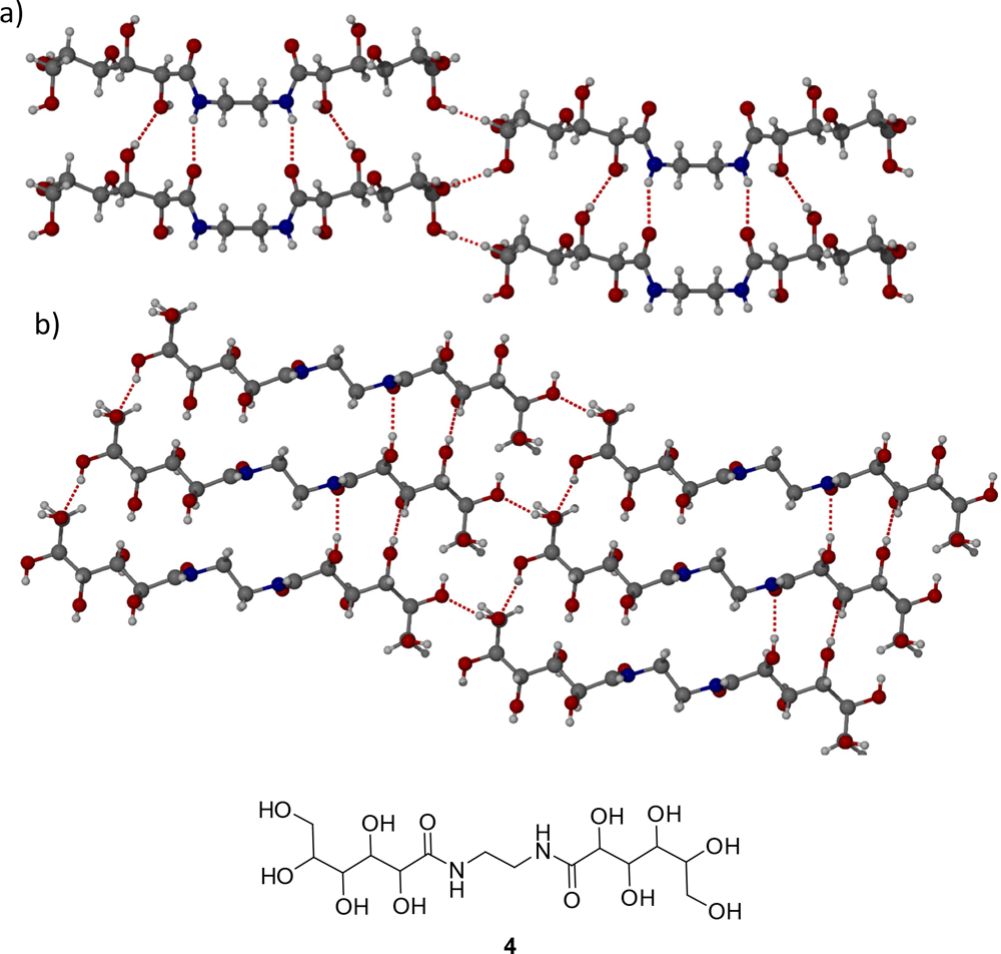
X-ray crystal structure of *N,N′*-ethylene bis-l-gluconamide (**4**) showing the amide hydrogen bond chain in the (a) (100) and (b) (010) crystallographic planes. The chemical structure of *N,N′*-ethylene bis-l-gluconamide is shown.

While compound **1** is used in the commercial product, related amides have similar properties.^24−26^ For comparison, and to further probe the formation of intermolecular amide NH···O interactions observed in form II as opposed to the intramolecular interaction in form I, a related gluconamide *N,N′*-ethylene bis-l-gluconamide (**4**) was prepared using the reported procedure.^24−26^ A solution of ethylenediamine in methanol with l-gulonic acid γ-lactone was refluxed under nitrogen to give a white powder of the diamide which was confirmed to be free of monoamide by proton nuclear magnetic resonance (^1^H NMR) spectroscopy. This material was dissolved in water, and methanol was added as an antisolvent which resulted in the formation of single crystals suitable for SXRD analysis (Figure 4). Powder X-ray diffraction (XRPD) established that the bulk material is phase pure and consistent with the pattern calculated from SXRD data (Supplementary Information Figure S5). The X-ray crystal structure is in the Sohncke space group *C*2 consistent with a single enantiomer of the gluconamide and contains one *N,N′*-ethylene bis-l-gluconamide molecule in the asymmetric unit. The carbonyl oxygen atom is slightly disordered over two positions in a ratio of 9:1. The *N,N′*-ethylene bis-l-gluconamide is situated on a 2-fold axis passing through the central C–C bond, and hence both halves of the molecule are equivalent. The hydrogen-bonding network is similar to form II of **1** with an intermolecular hydrogen bonded amide chain, with a similar N···O distance of 2.7991(18) Å. Each molecule of *N,N′*-ethylene bis-l-gluconamide is hydrogen bonded to 10 neighbors, showing that the system forms an extensive hydrogen bonded network similar to **1**. The range of O···O distances between alcohol groups is 2.6910(13)–2.8061(15) Å. The structures of form II of **1** and of **4** imply a propensity to form protein-like aggregates and possibly gel-forming behavior analogous to a range of amide-based low-molecular-weight gelators.^31,32^

### Amino Acid Salt Screen

Amino acids were selected as small-molecule models of the protein structure present in hair; however, the amino acids of course do not completely model the amide-linked polymeric structure bonding, the complex hydrogen bonding found in proteins, and the long chain length. Nevertheless, the amino acid substituents may give some insight into the interactions of hair care components with the amino acid residues in hair protein. COSMOquick was selected to screen the amino acids for cocrystal formation with each of **1**, 3AP, and GLA screened separately. COSMOquick uses the Conductor like Screening Model for Real Solvents (COSMO-RS) method to create charge density surfaces. The charge density surfaces describe each molecule, and the surfaces of two different molecules can be used to calculate interaction energies including the excess enthalpy of mixing (Δ*H*_mix_).^33^ The neutral forms of **2** and **3** were used in COSMOquick because the software is only parameterized for a limited selection of precalculated ionic species.^34,35^ The components **1**, 3AP, and GLA were screened individually as they exist as separate species when dissolved in water, and the experiment aimed to understand the interactions of the amino acids with each individual component. The results of the COSMOquick screen (Table S1) showed that **1** and GLA have similar interactions with amino acids and the combination of **1** or GLA with l-lysine gives the most negative Δ*H*_mix_. The top four amino acids (Table S1) with the most favorable excess enthalpy of mixing for each component were selected for cocrystal or salt screening. A range of experiments aimed at the preparation of cocrystals were performed with the selected systems including the use of mechanochemistry with grinding, liquid-assisted grinding, and a range of solution crystallizations including evaporation, antisolvent, and cooling crystallizations. However, no new cocrystals or salts of **1**, 3AP, or GLA with amino acids were produced, although a new dimethyl sulfoxide solvate of cysteic acid was formed (Figure S1). In the absence of amino acid cocrystals, the combination of amide **1**, 3AP, and GLA with small molecules that mimic the amino acid substituents was examined. The small molecules were initially screened with COSMOquick (Table 2), and in all cases, the molecules selected to mimic the amino acid substituent groups showed more favorable Δ*H*_mix_ with **1**, 3AP, and GLA, compared to the corresponding amino acids. The substituent group mimics were, therefore, experimentally screened with **1**, GLA, and 3AP.

**Table 2:** Potential Excess Enthalpy of Mixing of the Three Components of the Haircare Solution with the Molecules That Mimic the Substituent Groups of the Amino Acids Calculated Using COSMOquick^33^

hydroxypropyl-l-gluconamide		l-gluconic acid		3-amino-1-propanol	
co-former	Δ*H*_mix_	co-former	Δ*H*_mix_	co-former	Δ*H*_mix_
ethylenediamine	–4.764	ethylenediamine	–5.073	sulfuric acid	–9.654
guanidine	–2.604	guanidine	–2.773	oxalic acid	–5.405
				methanesulfonic acid	–3.919

**Figure 5:**
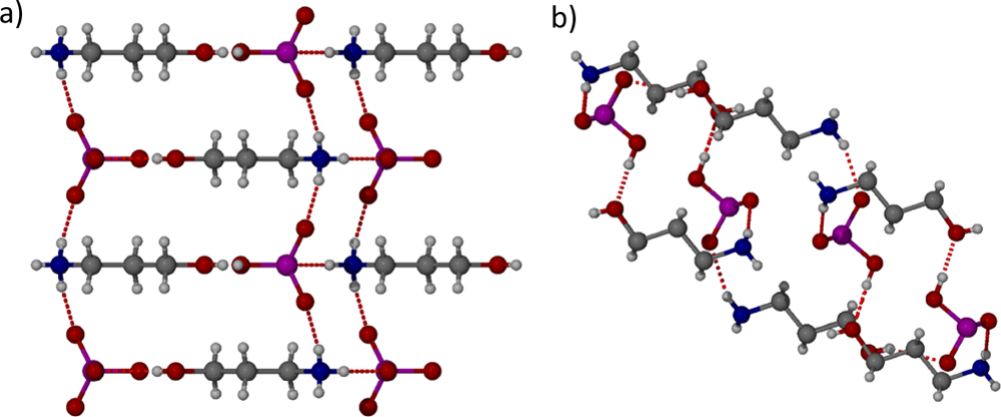
X-ray crystal structure of **2**·HSO_4_^–^ showing the hydrogen bonding interactions in the (a) (100) and (b) (010) crystallographic planes.

Sulfuric acid (H_2_SO_4_) was chosen to mimic the sulfonic acid substituent group of cysteic acid because of the structural similarity and the large negative excess enthalpy of mixing with 3AP observed in the COSMOquick screen (Table 2). A slight excess of sulfuric acid (H_2_SO_4_) was slowly added to 3AP. The vial was sealed, and after 15 days, small plate crystals had formed which were analyzed by SXRD. The structure was found to be a 1:1 salt 3-hydroxypropylammonium hydrogen sulfate (**2**·HSO_4_^–^) (Figure 5). Cation **2** forms intermolecular hydrogen bonds with five different hydrogen sulfate anions, with three hydrogen bonds forming between the N–H bonds and the S=O/S–O^–^ oxygen atoms with O···N distances varying from 2.8486(13) to 2.868(2) Å. One hydrogen bond forms between the O–H group of **2** and a sulfate oxygen atom with an O···O distance of 2.8250(19) Å. The fifth hydrogen bond forms between the hydrogen sulfate OH group and the hydroxyl oxygen atom of the **2** with an O···O distance of 2.519(2) Å, which is similar to a comparable structure of a sulfate anion with 4-hydroxyanilinium which has an O···O distance of 2.642(2) Å.^36^

**Figure 6:**
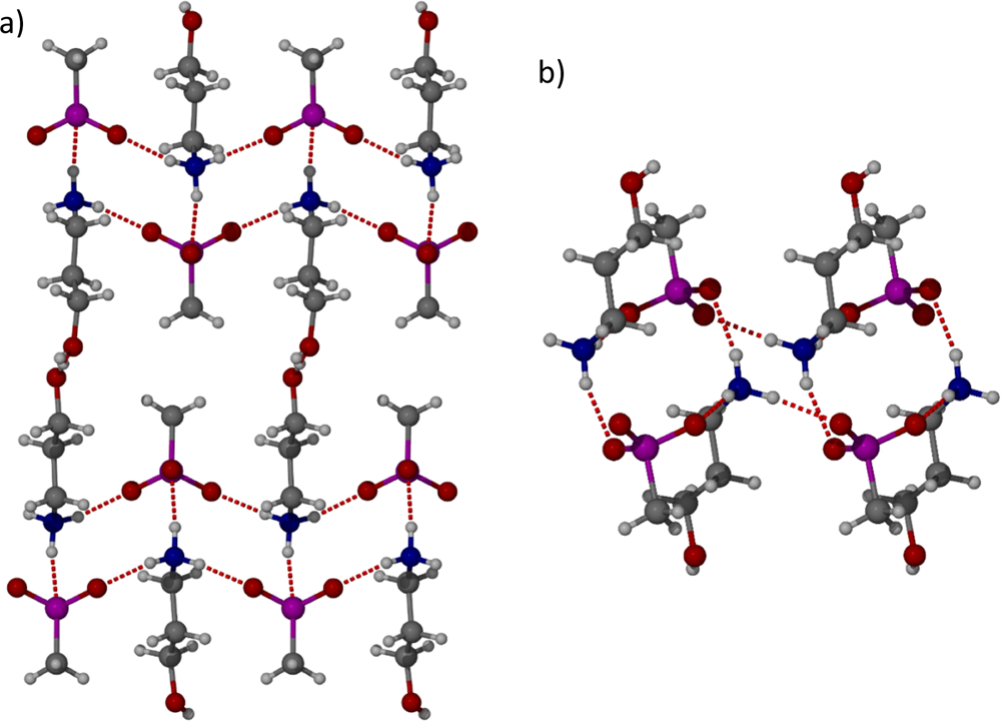
X-ray crystal structure of **2**·CH_3_SO_3_^–^ in the (a) (100) and (b) (101) crystallographic planes.

Methane sulfonic acid (CH_3_SO_3_H) was identified as a better model for cysteic acid compared to sulfuric acid, because of CH_3_SO_3_H being more structurally similar to cysteic acid. 3AP was added to a solution of CH_3_SO_3_H, and the temperature of the vial increased which was attributed to proton transfer. The system was then stored at 3 °C resulting in the formation of a white precipitate which was used as a seed crystal to produce a single crystal suitable for SXRD analysis. The system was found to be the salt **2**·CH_3_SO_3_^–^ (Figure 6) formed from two independent ionic pairs. The ammonium moiety interacts similarly with all three of the N–H bonds interacting with S=O/S–O^–^ oxygen atoms. The key difference between the CH_3_SO_3_H and H_2_SO_4_ salt structures is that the alcohol group of **2** no longer forms hydrogen bonds with any S=O/S–O^–^ or SOH oxygen atoms; instead it only forms hydrogen bonds with alcohol groups on other cations of **2** forming a repeating chain of alcohol groups. The change in the OH hydrogen bonding pattern can be attributed to the lack of SOH groups limiting the hydrogen bond donor potential. The hydrogen bond between alcohol groups is quite strong with the O···O distance alternating between 2.769(11) and 2.778(10) Å. The hydrogen bond lengths between S=O and NH are similar to the lengths observed with the **3**·HSO_4_^–^ structure with the O···N distances varying from 2.832(11), 2.829(11), 2.899(14), 2.821(11), 2.901(14), and 2.850(11) Å.

The **2**·HSO_4_^–^ and **2**·CH_3_SO_3_^–^ structures represent a plausible model for how cysteic acid could interact with **2**. From the two salt structures, it can be speculated that **2** could interact with two cysteic acid residues and produce a bridging interaction across the ammonium moiety. The bridging interaction between two cysteic acid residues with **2** could help strengthen damaged hair in a similar way to the original disulfide bridge which was present before the hair was damaged. The alcohol group could also interact with amino acids with substituent groups containing an alcohol group such as serine and threonine.

**Figure 7:**
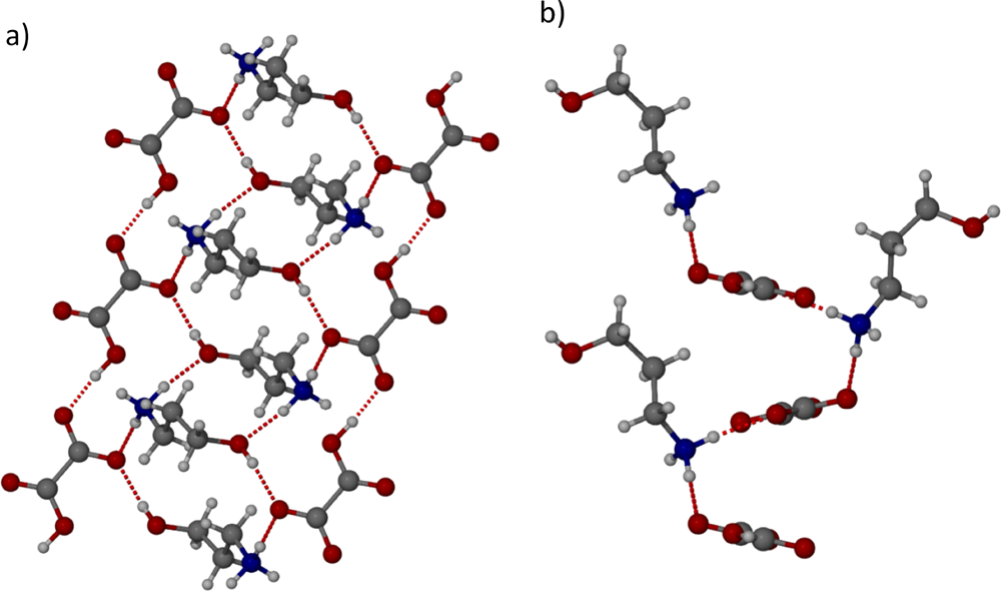
X-ray crystal structure of **2**·HC_2_O_4_^–^ in the (a) (010) and (b) (100) crystallographic planes.

Oxalic acid (C_2_H_2_O_4_) was selected to mimic the glutamic acid and aspartic acid because of the structural similarity of C_2_H_2_O_4_ with the substituent group of the amino acids and the large negative excess enthalpy of mixing from the COSMOquick screen (Table S2). C_2_H_2_O_4_ was dissolved in ethanol and 3AP was added which resulted in the formation of crystals. The crystals were analyzed by SXRD which determined the structure to be **2**·HC_2_O_4_^–^ (Figure 7). In the crystallization experiment, C_2_H_2_O_4_ was in excess with over four molecules of oxalic acid per one molecule of 3AP to encourage the formation of a 1:1 stoichiometric salt. The structure of **2**·HC_2_O_4_^–^ shows that only one of the carboxylic acid groups of the oxalic acid is deprotonated to give a hydrogen oxalate anion. The OH group on 3AP forms two hydrogen bonds, one via the hydrogen atom to the carboxylate anion side of HC_2_O_4_^–^ with an O···O distance of 2.7040(13) Å and the other via the oxygen atom to an NH group on another cation of **2** with an O···N distance of 2.8049(15) Å. The NH_3_^+^ group of **2** forms two hydrogen bonds to the carboxylate anions of two different C_2_HO_4_^–^ atoms with standard O···N distances of 2.7857(14) and 2.8414(15) Å. The HC_2_O_4_^–^ anions form a repeating hydrogen bonded chain from the OH of one HC_2_O_4_^–^ to the CO on another, and the O···O distance is very short at 2.5793(12) indicating that it is a very strong bond (Figure 7a).

**Figure 8:**
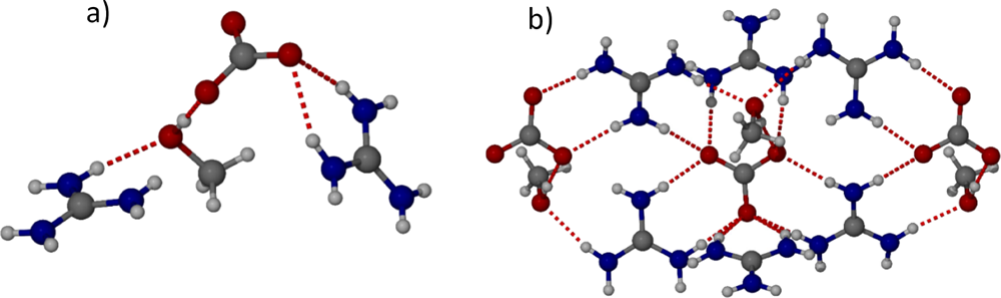
X-ray crystal structure of guanidinium carbonate methanol solvate. (a) Asymmetric unit and (b) extended packing.

Guanidine carbonate was chosen to mimic the interaction of the substituent group of arginine with **3**. l-Gulonic acid γ-lactone was suspended in methanol with guanidine carbonate, and the system was heated to reflux. The reaction produced a white powder suspended in a yellow solution. The white powder was separated by filtration and found to be guanidine carbonate by Fourier transform infrared (FTIR) spectroscopy. The yellow solution was sealed for 3 days which resulted in the formation of two different types of crystals. Both types of crystals were analyzed via SXRD with one identified as a previously characterized structure of guanidine carbonate (GUANCB)^37^ and the other proved to be a new methanol solvate of guanidine carbonate (Figure 8). The empirical formula of the methanol solvate contains two guanidine cations, one carbonate anion, and one methanol molecule. The GUANCB guanidine carbonate structure contains three R_2_^2^(8) hydrogen bonding motifs formed around one guanidine cation with three carbonate anions and six R_2_^2^(8) hydrogen bonding motifs formed around one carbonate anion with six guanidine cations.^37^ The O···N distance of hydrogen bonds in GUANCB varies from 2.704 to 3.189 Å. In the methanol solvate structure, the methanol molecule hydrogen bonds strongly to the carbonate with an O···O distance of 2.635(2) Å. The strong methanol to carbonate hydrogen bond disrupts the bonding motifs found in the original GUANCB structure, and the disruptions cause one of the hydrogen bonds between carbonate and guanidine to weaken and lengthen to 3.261(2) Å.

### Supramolecular Gelation Properties of Hydroxypropyl-l-gluconamide

**Figure 9:**
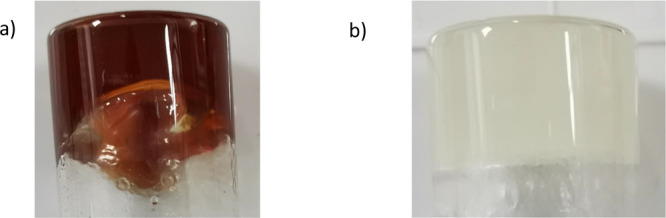
Images of the two supramolecular gels of **1** with (a) aniline and (b) benzyl alcohol.

A polymorph screen was performed on **1** with a range of 26 solvents based on covering the majority of the 15 solvent groups described by Gu et al.^38^ The screen involved making up 2 weight percent (wt %) solutions, heating to the boiling point, sonicating, and then leaving them to cool. The screen (Table 3) did not lead to any further polymorphs, but the system with aniline formed a supramolecular gel and the system with benzyl alcohol formed a partial gel.^39^ To form a gel of **1** in benzyl alcohol, the concentration was increased to 5 wt %, and the same process was repeated which resulted in a gel. The **1** aniline gel is translucent and has a dark orange color, and the **1** benzyl alcohol gel is opaque and has a cloudy white appearance (Figure 9). A gel forms by trapping solvent molecules in place with an elastic cross-linked network, forming a viscoelastic solid-like material.^40^ In the case of supramolecular gels, the cross-linked network is formed from the self-aggregation of low-molecular-weight gelators held together by intermolecular interactions.^31,41−44^ For a gel fiber to form the intermolecular interactions need to be strong and directional to produce one-dimensional chains, these chains form the primary structure of the gel.^45−47^ The secondary structure involves the aggregation of the molecular chain into fibers which then entangle to form the gel network which is classified as the tertiary structure.^31,48^ Gel formation represents an interesting result in this case implying supramolecular fiber formation and is consistent with the amide hydrogen bonded chains observed in the structures of form II of **1** and in **4**.

**Table 3:** Results of the Polymorph Screen of **1** with a Range of Solvents at 2 wt %^a^

solvent	result	solvent	result
1,4-dioxane	ND	ethylene glycol	P
acetic acid	S	formic acid	S
acetone	ND	hexane	ND
acetonitrile	ND	methanol	S
aniline	G	morpholine	S
benzene	ND	*N,N*-dimethylacetamide	S
benzyl alcohol	PG	nitromethane	ND
chlorobenzene	ND	*N*-methyl pyrrolidone	S
chloroform	ND	pyridine	S
diethyl ether	ND	tetrahydrofuran	ND
diethylamine	ND	toluene	ND
ethanol	P	triethylamine	S
ethyl acetate	ND	water	S

a G = gel, PG = partial Gel, S = solution, ND = not dissolved, and P = precipitate.

Systematic testing demonstrated that sonication after heating is essential for the formation of the **1** benzyl alcohol gel but is not necessary for the **1** aniline gel, but it does increase the rate of gel formation. The need for sonication suggests that the gel fibers in benzyl alcohol are not the most thermodynamically stable product and sonication induces the formation of a kinetically metastable state.^49−51^ The critical gelling concentration of **1** in aniline is 0.5 wt %, with a concentration of 0.4 wt % and below forming partial gels. For benzyl alcohol, the critical gel concentration is 4.5 wt %. The lower critical gelling concentration of **1** in aniline shows that it is a more potent gelator in this unusual solvent.

To investigate the structural characteristics of solvent that promote gels of **1** as the gelator, a second gel screen was performed with a range of aniline and amine derivatives (Scheme S1). The gel screen is summarized in Table S2. Three gels formed with **1** in 2,4-dimethylaniline, 3,4-difluoroaniline, and 4-butylaniline, and four partial gels formed with 2,6-dimethylaniline, 2-methoxyaniline, 3,5-dimethylaniline, and *N*-methylaniline. All of the aniline derivatives apart from *N,N*-dimethylaniline formed gels or partial gels. It is possible that *N,N*-dimethylaniline does not form a gel or partial gel because it does not contain any N–H bonds available to undergo hydrogen bonding. All of the aliphatic amine derivatives do not form any gels or partial gels. Similarly, neither benzylamine nor cyclohexylamine formed a gel or partial gel indicating that the aromatic amine group is essential for the formation of the gel network. Therefore, the gel screen indicates that a phenyl-derived group that is directly connected to a primary or secondary amine group is required for gel formation to take place.

#### Rheology

The viscoelastic properties of a range of different concentrations of the **1** aniline gel were assessed by oscillatory rheology.^52,53^

**Figure 10:**
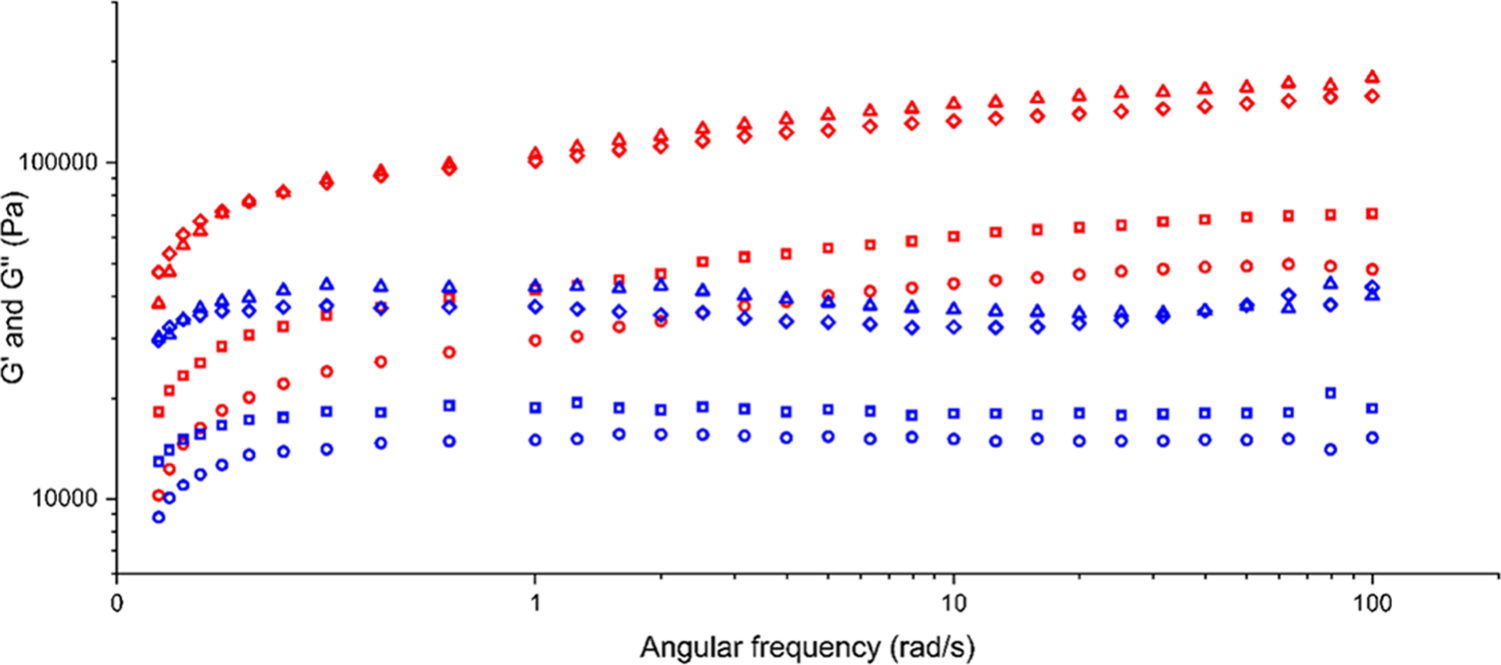
Oscillatory frequency sweep for different concentrations of **1** in aniline at 10 Pa. *G*′ is shown in red, and *G*″ is shown in blue. The different concentrations are 0.75 wt % (circle), 1 wt % (square), 1.5 wt % (triangle), and 2 wt % (diamond).

**Figure 11:**
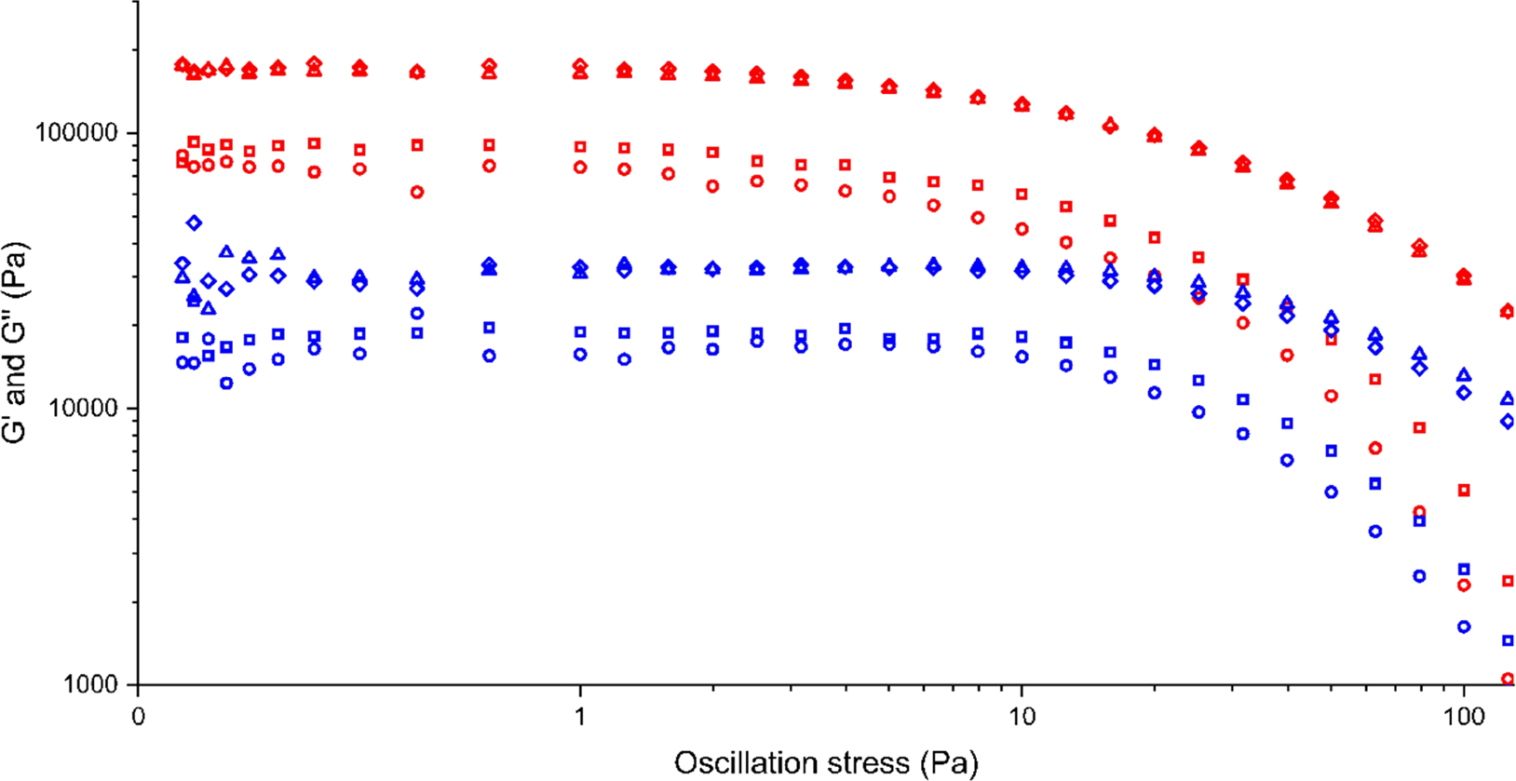
Oscillatory stress sweeps for a range of different concentrations of **1** in aniline at 10 rad/s. *G*′ is shown in red, and *G*″ is shown in blue. The different concentrations are 0.75 wt % (circle), 1 wt % (square), 1.5 wt % (diamond), and 2 wt % (triangle).

The oscillatory frequency sweep at a constant oscillatory stress of 10 Pa confirmed that *G*′ and *G*″ do not change with frequency and *G*′ is at least one magnitude higher than *G*″ for all gels (Figure 10). The oscillatory frequency sweep shows that the gel is strongest around 1.5–2 wt % because of the higher *G*′ and *G*″ values compared to the lower concentrations (Figure S2). The oscillatory stress sweep involves testing the sample at a constant angular frequency of 10 rad/s with increasing oscillatory stress. The gel strength increases with concentration, reaching a plateau at 1.5–2 wt % (Figure 11).

#### Xerogel Analysis

**Figure 12:**
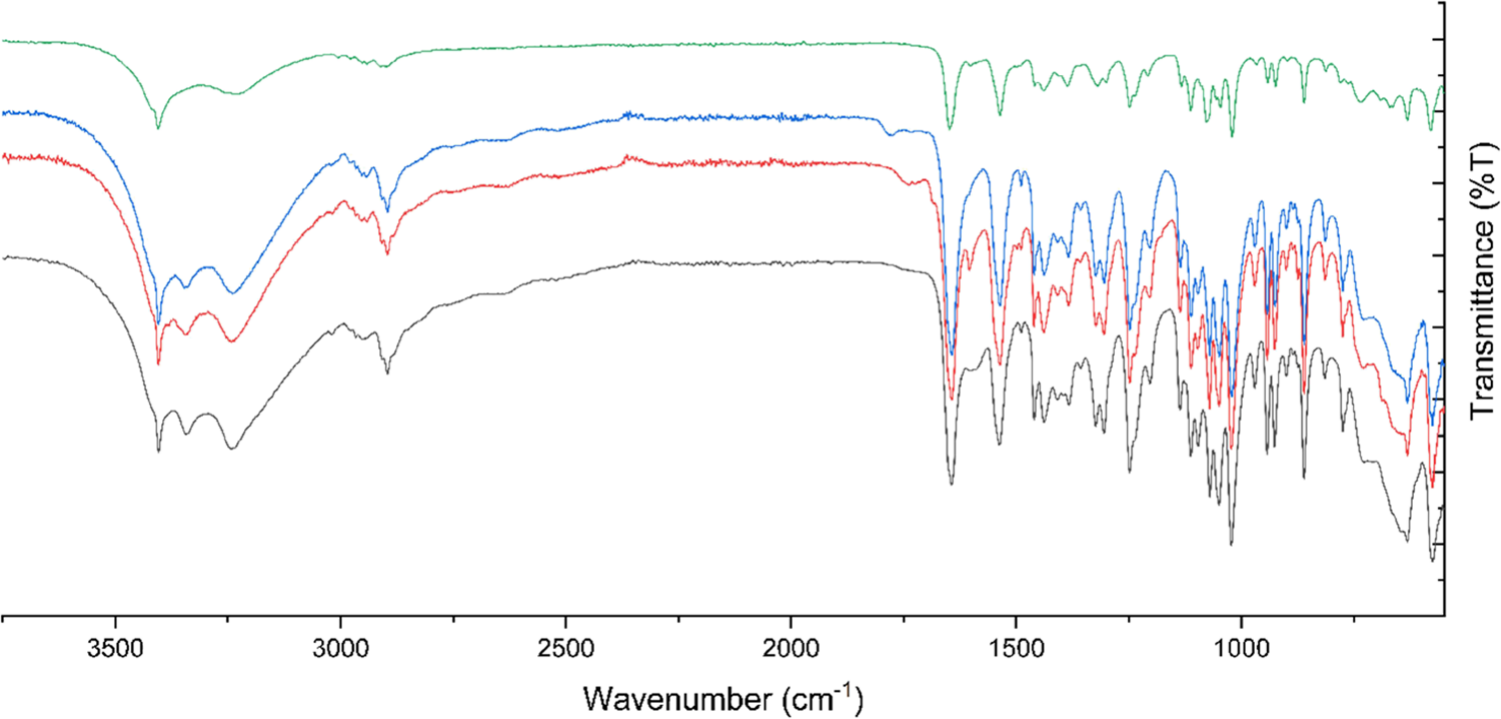
FTIR spectra of **1** form I (black), form II (green), **1** aniline xerogel (red), and **1** benzyl alcohol xerogel (blue).

Xerogels of a 1 wt % **1** aniline gel and a 5 wt % **1** benzyl alcohol gel were formed by leaving the gels in an open vial allowing the solvent to evaporated. The xerogels were initially analyzed by FTIR spectroscopy and compared to the FTIR spectra of both polymorphs of **1**. The FTIR spectra (Figure 12) show that the xerogel is surprisingly identical to form I of **1** and establishes that **1** has not been chemically altered or decomposed. The structure implies that the gel fiber structure may be similar to the crystal structure of form I which is surprising given that form I lacks an amide hydrogen bonded chain. However, the crystal structure may be significantly affected by solid form changes during the drying process and hence may not be representative of the gel structure.^54^

**Figure 13:**
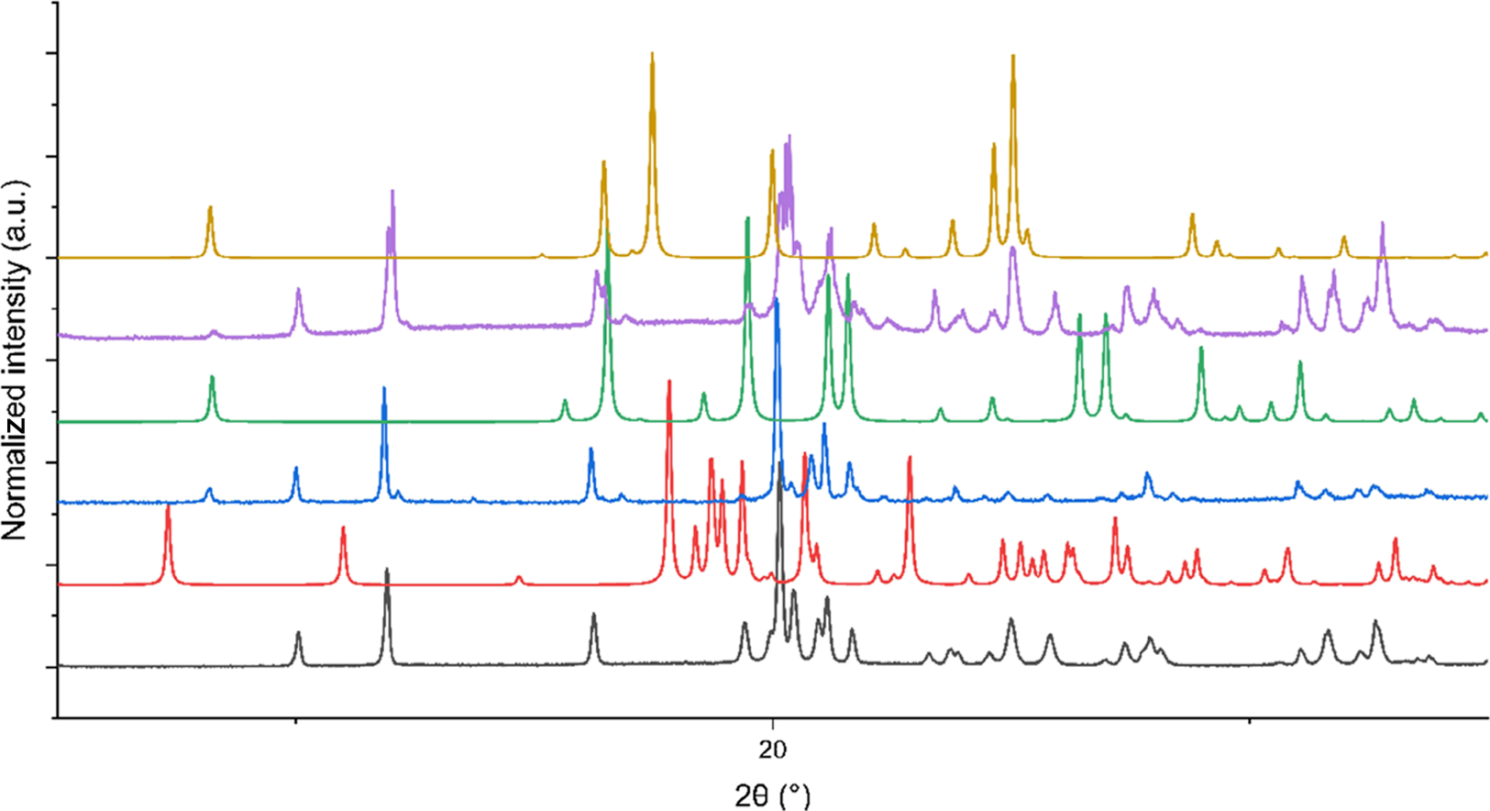
XRPD patterns of **1** form I (black), **1** form II (red), **1** aniline xerogel (blue), and **1** benzyl alcohol xerogel (purple). The calculated patterns from published crystal structures of aniline (green) and benzyl alcohol (orange) are shown.^55,56^

The xerogels of the **1** aniline and benzyl alcohol gels were analyzed by XRPD. The XRPD patterns (Figure 13) of the two xerogels are very similar to each other, and the majority of their peaks match the peaks from **1** form I. The XRPD is consistent with the FTIR data suggesting that the gel fiber is structurally similar to form I. A few extra peaks are observed which correlate with the calculated XRPD pattern of the frozen solvent, suggesting that the xerogel is not completely dry.

The solution ^1^H NMR spectra (Figures S3 and S4) of the aniline xerogel and pure **1** are identical indicating that **1** has not reacted with the aniline or decomposed into the gluconate salt. The spectra of the benzyl alcohol xerogel, however, show partial hydrolysis with the sample containing 88% **1** and 12% gluconate salt. The partial hydrolysis may have been caused by the heating step to form the gel, the presence of moisture or during the slow evaporation of the solvent.

**Figure 14:**
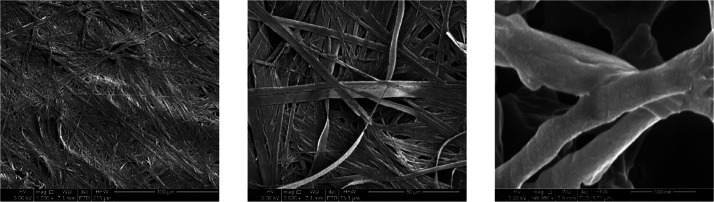
SEM micrographs of the dried xerogel of **1** aniline at 1 wt %.

**Figure 15:**
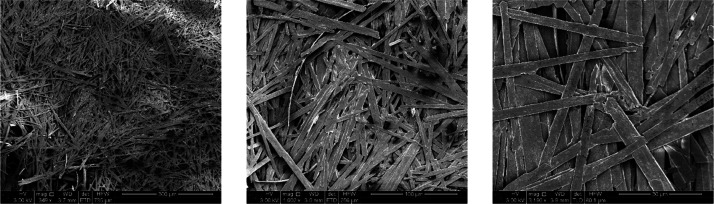
SEM micrographs of the dried xerogel of **1** benzyl alcohol at 5 wt %.

The xerogels were analyzed via scanning electron microscopy (SEM) to visualize the fibers formed in the gels. The SEM images of the **1** aniline xerogel (Figure 14) show a fibrillar network which is characteristic of gels. The gel fibers are relatively large with a width of between 0.35 and 2 μm. The SEM images of the **1** benzyl alcohol xerogel (Figure 15) do not show the characteristic gel fibers; instead they show small plank-shaped crystals with a larger width of 3.5–7.5 μm. These crystalline-appearing fibers features suggest that the gel fibers in the benzyl alcohol gel are not very stable (as indicated in the sonication study), and they may crystallize as part of the drying process.

## Conclusions

Two polymorphic forms of **1** were analyzed by SXRD revealing an extensive network of hydrogen bonding taking place between the alcohol groups, suggesting that **1** could form an extensive hydrogen bonding network with amino acids present in hair fibers. One of the key differences between the two polymorphs is the NH group either forming an intramolecular hydrogen bond in form I and an intermolecular hydrogen bond in form II. The gluconamide *N,N′*-ethylene bis-l-gluconamide was crystallized and analyzed by SXRD showing a similar extensive network of hydrogen bonds from the gluconic acid part of the molecule as seen in the crystal structures of both polymorphs of **1**. The *N,N′*-ethylene bis-l-gluconamide also shows the same intermolecular hydrogen bond between amide units as observed in form II of **1**.

A COSMOquick screen was performed to identify the most energetically favorable cocrystals or salts that could form between **1**, **2**, and **3**, with the amino acids present in hair. The most energetically favorable combinations of **1**, **2**, and **3** with amino acids were screened experimentally using a variety of cocrystallization techniques, but no new structures were found. To simplify the potential interactions, smaller molecules were selected to mimic the amino acid substituent groups. The small molecules were screened using COSMOquick which showed that the systems with the small molecules were more energetically favorable compared to the original amino acids. No cocrystal or salt structures were identified with **1** or **3**. A new methanol solvate of guanidine carbonate was isolated and suggests potential interactions between the carboxylate anion of **3** and a guanidinium cation present in arginine. Three new salt structures of **2** with sulfuric acid, methane sulfonic acid, and oxalic acid were determined, suggesting potential interactions of **2** with the amino acids cysteic acid, aspartic acid, and glutamic acid.

Hydroxypropyl-l-gluconamide was found to act as a supramolecular gelator in benzyl alcohol, aniline, and a range of aniline derivatives. Sonication was required to form the **1** benzyl alcohol gel but it was not required to form the **1** aniline gel, indicating the **1** benzyl alcohol gel fibers are in a metastable state. The gel fibers of the xerogels were analyzed via SEM which showed that the **1** aniline gel displayed characteristic gel fibers. However, the SEM of the **1** benzyl alcohol gel showed small crystals had formed indicating that the metastable gel fibers had recrystallized when the solvent was removed.

Work is ongoing regarding the mechanism of action of hydroxypropyl-l-gluconamide and hydroxypropylammonium gluconate in hair strengthening but this work suggests that strongly hydrogen bonded salt bridges may play a role as a substitute for damaged disulfide bridges. The gelation properties of hydroxypropyl-l-gluconamide are surprising and may indicate a tendency of the compound to aggregate and perhaps coat hair fibers, imparting volume and strengthening.

## Experimental Section

### Materials

FiberHance bm solution was supplied by Ashland LLC. All other materials were purchased either from Merck or Thermo Fisher Scientific and were used without further purification.

### Analytical Methods

^1^H and ^13^C solution NMR spectra were recorded using a Varian Mercury-400 spectrometer, operating at 400 MHz for ^1^H and 100 MHz for ^13^C, and chemical shifts were reported in ppm (δ) and referenced to residual protic solvent.

FTIR spectra were measured with a PerkinElmer 100 FT-IR Spectrometer with an μATR attachment. Data were recorded at a resolution of 4 cm^–1^ for 12 scans over a range of 4000 to 550 cm^–1^.

XRPD measurements were performed using a Bruker D8 X-ray diffractometer (Billerica, Massachusetts) with CuKα radiation (1.54187 Å) and an acceleration voltage and current of 40 kV and 40 mA, respectively. The samples were scanned in reflectance mode between 3° and 60° 2θ with a scan rate of 0.01583° 2θ/s and a step size of 0.02°.

Elemental analysis was performed by the University of Durham service using an Exeter CE-440 Elemental Analyzer.

The X-ray single crystal data for all compounds have been collected using λMoKα radiation (λ = 0.71073 Å) on Bruker D8Venture diffractometers at various configurations (Photon100 CMOS detector, IμS-microsource, Helois focusing mirrors, compounds **1**_I/Photon III MM C14 CPAD detector, IμS-III-microsource, Helois focusing mirrors compounds **1D**, **4**, LCA-DMSO, **2**-HSO_4_, **2**-MeSO_3_, GCB-MeOH/Photon III MM C7 CPAD detector, IμS-microsource, Helois focusing mirrors, compounds **1**_II, **2**-HC_2_O_4_) equipped with Cryostream (Oxford Cryosystems) open-flow nitrogen cryostats at a temperature of 120.0(2)K. All structures were solved by direct methods and refined by full-matrix least squares on *F*^2^ for all data using Olex2^57^ and SHELXTL^58^ software. All nonhydrogen atoms were refined anisotropically, and hydrogen atoms in most of the structures were found in difference Fourier maps and refined in isotropic approximation. Hydrogen atoms in the twinned structure of **2·**CH_3_SO_3_^–^ (TWINABS/HKLF 5 refinement) and those of CH_2_-groups in the structure **1** form 2 (*Z*′ = 2) were placed in the calculated positions and refined in riding mode. Absolute structures of all chiral compounds (except l-cysteic acid dimethylsulfoxide solvate, where it was determined from experimental data by anomalous dispersion effects) were assigned on the basis of known configurations of starting materials.

Oscillatory rheometry measurements were performed using a TA Instruments AR 2000, on a rough Peltier top plate, with a 25 mm rough plate geometry and 2.5 mm gap, and a bottom plate containing a small well with a diameter of 26 mm and a depth of 2.5 mm. Samples were prepared by heating preprepared gels until they dissolved. A portion of the solution was then pipetted into the well of the rheometer plate, which was set to maintain a temperature of 20 °C throughout the formation and analysis of the gels. The solution was covered with a watch glass during gel formation to limit evaporation. The gels were allowed to form over 10 min before analysis. Oscillatory frequency sweep experiments were performed with a constant applied stress of 10 Pa, and oscillatory stress sweep experiments with a constant angular frequency of 10 rad/s.

SEM samples were prepared on silicon wafers, dried in air for 2 days, and coated with 2.5 nm of platinum using a Cressington 328 Ultra High Resolution EM Coating System. The images were obtained using a FEI Helios Nanolab 600 microscope.

### Crystallization of **1** Form I

FiberHance bm solution (2 mL) was left to slowly evaporate. After 1 week lath shaped crystals formed. Analysis calc. of C_9_H_19_NO_7_: C 42.68, H 7.56, 5.54%, found: C 42.60, H 7.53, N 5.46%; FTIR (ν/cm^–1^): 3404, 3342, 3239, 2895, 1643, 1538, 1460, 1439, 1383, 1324, 1305, 1248, 1238, 1203, 1137, 1113, 1097, 1070, 1050, 1022, 971, 943, 927, 861, 775, 632, and 576. ^1^H NMR (400 MHz, D_2_O) δ 4.18 (d, *J* = 3.6 Hz, 1H), 3.96 (t, *J* = 3.2 Hz, 1H), 3.72–3.68 (m, 2H), 3.64–3.61 (m, 2H), 3.56–3.50 (m, 2H), 3.2 (td, *J* = 6.9, 2.8 Hz, 2H), 1.66 (p, *J* = 6.7 Hz, 2H). Crystal data: C_9_H_19_NO_7_*M* = 253.25 g mol^–1^, 0.28 × 0.18 × 0.11 mm^3^, monoclinic, space group *P*2_1_, *a* = 4.6468(2) Å, *b* = 13.9198(7) Å, *c* = 8.9183(5) Å, α = 90°, β = 101.1403(19)°, γ = 90°, *V* = 565.99(5) Å^3^, *Z* = 2, *D*_C_ = 1.486 g cm^–3^, *F*_000_ = 272.0, 12,795 reflections collected, 3305 unique (*R*_int_ = 0.0325). Final GooF = 1.037, *R*_1_ = 0.0325 (3305 reflections with *I* ≥ 2σ(*I*)), w*R*_2_ = 0.0823 (all data), 230 parameters, 1 restraint, μ = 0.128 mm^–1^.

### Crystallization of d-GLA **1** Form I

d-Gluconic acid solution was mixed with 3AP forming a viscous yellow solution. The solution was left to slowly evaporate forming colorless plank crystals. FTIR (ν/cm^–1^): 3404, 3342, 3239, 2895, 1643, 1538, 1460, 1439, 1383, 1324, 1305, 1248, 1238, 1203, 1137, 1113, 1097, 1070, 1050, 1022, 971, 943, 927, 861, 775, 632, and 576. Crystal data: C_9_H_19_NO_7_*M* = 253.25 g mol^–1^, 0.29 × 0.1 × 0. mm^3^, monoclinic, space group *P*2_1_, *a* = 4.64620(10) Å, *b* = 13.9212(4) Å, *c* = 8.9163(3) Å, α = 90°, β = 101.1335(11)°, γ = 90°, *V* = 565.86(3) Å^3^, *Z* = 2, *D*_C_ = 1.486 g cm^–3^, *F*_000_ = 272.0, 13,525 reflections collected, 3268 unique (*R*_int_ = 0.0403). Final GooF = 1.021, *R*_1_ = 0.0366 (3268 reflections with *I* ≥ 2σ(*I*)), w*R*_2_ = 0.0934 (all data), 230 parameters, 1 restraint, μ = 0.128 mm^–1^.

### Crystallization of **1** Form II

**1** (50 mg, 0.20 mmol) was suspended in ethanol (150 mL). Aniline (100 μL, 1.11 mmol) was added, and the mixture was refluxed with stirring for 30 min. The solution was left to evaporate in a round bottom flask for 5 months. A white solid with an orange tinge was formed. The solid (4.6 mg) was dissolved in ethanol (100 μL) and the solution was left to evaporate. Plank crystals of SXRD quality formed after 3 days. FTIR (ν/cm^–1^): 3405, 3239, 2895, 1648, 1536, 1459, 1439, 1386, 1320, 1300, 1248, 1237, 1208, 1134, 1113, 1107, 1047, 1021, 943, 924, 861, 813, 761, 763, 734, 670, 631, and 580. Crystal data: C_9_H_19_NO_7_*M* = 253.25 g mol^–1^, 0.15 × 0.05 × 0.01 mm^3^, monoclinic, space group *P*2_1_, *a* = 9.5157(4) Å, *b* = 5.0795(2) Å, *c* = 24.2667(10) Å, α = 90°, β = 96.4629(14)°, γ = 90°, *V* = 1165.48(8) Å^3^, *Z* = 4, *D*_C_ = 1.443 g cm^–3^, *F*_000_ = 544.0, 19,083 reflections collected, 6127 unique (*R*_int_ = 0.0501). Final GooF = 1.028, *R*_1_ = 0.0514 (6127 reflections with *I* ≥ 2σ(*I*)), w*R*_2_ = 0.1110 (all data), 363 parameters, 1 restraint, μ = 0.124 mm^–1^.

### Cosmoquick Screen

COSMOquick version 1.7 (COSMOlogic GmbH & Co. KG, Leverkusen, Germany) was used to calculate the excess enthalpy of mixing for each component of the haircare mixture with amino acids and a range of amino acid mimics.^33^

### **2**·HSO_4_^–^ Salt

Sulfuric acid (80 μL, 1.5 mmol) was slowly added to 3AP (50 μL, 0.65 mmol). The sample released a vapor and was hot to the touch upon the addition of sulfuric acid. The vial was left sealed for 15 days until small plate-shaped crystals formed. Crystal data: C_3_H_11_NO_5_S *M* = 173.19 g mol^–1^, 0.15 × 0.08 × 0.01 mm^3^, monoclinic, space group *P*2_1_/m, *a* = 5.3514(3) Å, *b* = 6.9661(4) Å, *c* = 9.6220(5) Å, α = 90°, β = 98.976(2)°, γ = 90°, *V* = 354.30(3) Å^3^, *Z* = 2, *D*_C_ = 1.623 g cm^–3^, *F*_000_ = 184.0, 6372 reflections collected, 1104 unique (*R*_int_ = 0.0377). Final GooF = 1.146, *R*_1_ = 0.0306 (1104 reflections with *I* ≥ 2σ(*I*)), w*R*_2_ = 0.0708 (all data), 83 parameters, 0 restraints, μ = 0.426 mm^–1^.

### **2·**CH_3_SO_3_^–^ Salt

3AP (50 μL, 0.65 mmol) was slowly added to methane sulfonic acid (90 μL, 1.39 mmol). Upon the addition of 3AP, the temperature of the vial increased, and white gas was released. The sealed vial was stored at 3 °C which resulted in the formation of a white precipitate after 2 h. The white precipitate was used as a seed crystal and added to a solution of 3AP (50 μL, 0.65 mmol), methane sulfonic acid (90 μL, 1.39 mmol), and ethanol (200 μL) and stored at 3 °C. After a few hours, plate crystals formed. Crystal data: C_4_H_13_NO_4_S *M* = 171.21 g mol^–1^, 0.21 × 0.07 × 0.01 mm^3^, monoclinic, space group *P*2_1_, *a* = 5.1527(2) Å, *b* = 21.5379(10) Å, *c* = 7.1287(3) Å, α = 90 °, β = 91.6578(19) °, γ = 90 °, *V* = 790.80(6) Å^3^, *Z* = 4, *D*_C_ = 1.438 g cm^–3^, *F*_000_ = 368.0, 18,888 reflections collected, 18,888 unique (*R*_int_ = 0.1040). Final GooF = 1.018, *R*_1_ = 0.0665 (18,888 reflections with *I* ≥ 2σ(*I*)), w*R*_2_ = 0.1757 (all data), 187 parameters, 1 restraint, μ = 0.372 mm^–1^.

### **2**·HC_2_O_4_^–^ Salt

Oxalic acid (50 mg, 0.56 mmol) was dissolved in ethanol (250 μL). 3AP (10 μL, 0.13 mmol) was added to the vial which resulted in the formation of plate crystals. FTIR (ν/cm^–1^): 3100, 1689, 1395, 1346, 1163, 779, 672, and 652. Crystal data: C_5_H_11_NO_5_*M* = 165.15 g mol^–1^, 0.11 × 0.1 × 0.02 mm^3^, monoclinic, space group *P*2_1_/*n*, *a* = 5.6912(4) Å, *b* = 7.1078(5) Å, *c* = 19.2926(14) Å, α = 90 °, β = 90.414(3) °, γ = 90 °, *V* = 780.40(10) Å^3^, *Z* = 4, *D*_C_ = 1.406 g cm^–3^, *F*_000_ = 352.0, 12,905 reflections collected, 2255 unique (*R*_int_ = 0.0450). Final GooF = 1.061, *R*_1_ = 0.0414 (2255 reflections with *I* ≥ 2σ(*I*)), w*R*_2_ = 0.0993 (all data), 144 parameters, 0 restraints, μ = 0.126 mm^–1^.

### Guanidine Carbonate Methanol Solvate

l-Gulonic acid γ-lactone (1 g, 5.6 mmol) and guanidine carbonate (2 g, 22.2 mmol) was suspended in methanol (10 mL). The mixture was degassed with nitrogen for 30 min and then refluxed under nitrogen for 3 h. The mixture was cooled, and it contained a white powder in a light yellow transparent solution. The solid powder was removed via filtration and identified by FTIR as guanidine carbonate. The yellow solution was left sealed for 3 days and crystals formed. The vial contained two different types of lath shaped crystal with one crystal identified as guanidine carbonate (GUANCB)^37^ and the other a new structure of guanidine carbonate methanol solvate. Crystal data: C_4_H_16_N_6_0_4_*M* = 212.23 g mol^–1^, 0.21 × 0.06 × 0.01 mm^3^, orthorhombic, space group *P*2_1_2_1_2_1_, *a* = 7.1149(3) Å, *b* = 11.6098(4) Å, *c* = 13.7967(5) Å, α = 90 °, β = 90 °, γ = 90 °, *V* = 1139.64(7) Å^3^, *Z* = 4, *D*_C_ = 1.237 g cm^–3^, *F*_000_ = 456.0, 20,652 reflections collected, 3319 unique (*R*_int_ = 0.0471). Final GooF = 1.103, *R*_1_ = 0.0415 (3319 reflections with *I* ≥ 2σ(*I*)), w*R*_2_ = 0.0985 (all data), 180 parameters, 0 restraints, μ = 0.107 mm^–1^.

### *N*,*N*′-Ethylene Bis-l-gluconamide

Ethylenediamine (0.53 mL, 9.8 mmol) was mixed with methanol (20 mL), and l-gulonic acid γ-lactone (2.852 g, 16.0 mmol) was added.^24^ The solution was refluxed with stirring under nitrogen for 2 h. A white powder forms during the reaction which was separated by filtration. Ten milligrams of the powder was dissolved in water (20 μL), methanol (20 μL) was added, and the sample formed crystals after a few hours. FTIR (ν/cm^–1^): 3289, 2933, 2879, 1642, 1538, 1434, 1315, 1077, 1043, and 878. Crystal data: C_14_H_28_N_2_O_12_*M* = 416.38 g mol^–1^, 0.21 × 0.17 × 0.12 mm^3^, monoclinic, space group *C*2, *a* = 9.7045(4) Å, *b* = 5.0273(2) Å, *c* = 18.1838(7) Å, α = 90 °, β = 90.9710(10) °, γ = 90 °, *V* = 887.01(6) Å^3^, *Z* = 2, *D*_C_ = 1.559 g cm^–3^, *F*_000_ = 444.0, 10,231 reflections collected, 2537 unique (*R*_int_ = 0.0260). Final GooF = 1.105, *R*_1_ = 0.0252 (2537 reflections with *I* ≥ 2σ(*I*)), w*R*_2_ = 0.0665 (all data), 187 parameters, 1 restraint, μ = 0.137 mm^–1^.

### l-Cysteic Acid Dimethylsulfoxide Solvate Synthesis

**1** (5.8 mg, 0.023 mmol) and cysteic acid monohydrate (4.3 mg, 0.023 mmol) were dissolved in dimethylsulfoxide (400 μL). Chloroform (1.2 mL) was vapor diffused into the solution resulting in the formation of small prism crystals. Crystal data: C_5_H_13_NO_6_S_2_*M* = 247.28 g mol^–1^, 0.45 × 0.34 × 0.14 mm^3^, monoclinic, space group *P*2_1_, *a* = 6.5483(3) Å, *b* = 7.9607(3) Å, *c* = 9.8718(4) Å, α = 90°, β = 93.5090(19)°, γ = 90 °, *V* = 513.64(4) Å^3^, *Z* = 2, *D*_C_ = 1.599 g cm^–3^, *F*_000_ = 260.0, 8225 reflections collected, 2839 unique (*R*_int_ = 0.0388). Final GooF = 1.056, *R*_1_ = 0.0337 (2839 reflections with *I* ≥ 2σ(*I*)), w*R*_2_ = 0.0881 (all data), 179 parameters, 1 restraint, μ = 0.523 mm^–1^.

### Gel Screening Procedure

The gelation behavior of **1** was initially tested in a range of solvents by producing a 2 wt % sample. The sample was sonicated for 1 min and then heated to the boiling point of the solvent using a heat gun in a sealed glass vial. The sample was then sonicated for 1 min and left to cool in an insulating wooden block. A similar process was followed for the amine and aniline derivative gel screen with 5 mg of **1** added to a vial and solvent added and heated to boiling point until it fully dissolves. If **1** had not started to dissolve by 1 wt % the sample was labeled as not dissolved.

### Sonication Study

To test if sonication is required for gel formation two 2 wt % solutions of **1** in aniline were made and heated to 120 °C for 5 min. One of the solutions was left to cool, and the other was sonicated for 1 min. The samples were then monitored visually for gelation. The same process was repeated for 5 wt % solutions of **1** in benzyl alcohol.

### Critical Gelling Concentration Study

To identify the critical gelling concentration of the gels, a 2 wt % solution of aniline was gelled using the previously described method. Then the wt % of the solution was gradually decreased with the addition of aniline and the gel formation method was repeated with the sample visually analyzed for gel formation. If a gel formed more aniline was added and the process was repeated until no gel or a partial gel formed and the last concentration to result in the formation of a gel was recorded as the critical gelling concentration. The process was repeated with a 5 wt % solution of **1** in benzyl alcohol.

### Xerogel Formation

To form the dried xerogels, a 2 wt % gel of **1** in 1 mL of aniline was formed in a small vial, and the lid was left open allowing the solvent to slowly evaporate over a few weeks leaving behind the xerogel. The same process was repeated with a gel of 5 wt % **1** in 1 mL of benzyl alcohol.

## Supporting Information Available

Electronic Supplementary Information (ESI) available: COSMOquick calculation results, X-ray structures, gel screen results, ^1^H NMR data and crystallographic information tables. Crystal structure have been deposited with the Cambridge Structural Database CCDC 2177450–2,177,458. Underlying data for IR, XRPD, rheology, and NMR experiments (PDF)
