# Supplementary material for: Understanding the Interaction of Gluconamides and Gluconates with Amino Acids in Hair Care
Source: Cryst Growth Des. 2022 Sep 20;22(10):6190–200. doi: 10.1021/acs.cgd.2c00753 (PMC9542698; doi:10.1021/acs.cgd.2c00753)
Supplement: Supplementary file 1 — cg2c00753_si_001.pdf [file cg2c00753_si_001.pdf]

Understanding the Interaction of Gluconamides and Gluconates in Hair Care

Luke I. Chambers[1], Dmitry S. Yufit,[1] Osama M. Musa[2], Jonathan W. Steed[1*]

1) Durham University, Department of Chemistry, Lower Mountjoy, Stockton Road, Durham, DH1 3LE, UK.
2) Ashland LLC, 1005 Route 202/206, Bridgewater, NJ 08807, USA.

*Corresponding author. Email address: jon.steed@durham.ac.uk

Supplementary Material

Table S1: The potential excess enthalpy of mixing of the 3 components of the haircare solution with all the amino acids in hair calculated using COSMOquick.[1]

Figure S1: The X-ray crystal structure of a DMSO solvate of *L*-cysteic acid.

Scheme S1: Aniline, benzyl alcohol and the aniline and amine derivatives used for the second gel screen.

Table S2: Gel screen of **1** with a range of aniline and amine derivatives with varying wt%. G = gel, PG = partial gel, S = solution, ND = not dissolved, P = Precipitate, VL = viscous liquid.

Figure S2: The variation of G' at 10 rad/s from the oscillatory frequency sweep for different concentrations of compound **1** in aniline.

Figure S3: The labelled diagram of **1** and the gluconate salt for NMR assignment.

Figure S4: The ^{1}H NMR spectrum of **1** (black), **1** aniline xerogel (red) and **1** benzyl alcohol xerogel (green). Two peaks are assigned based on the labelling in **Figure** to show the ratio of **1** to the salt decomposition product.

Table S3: Crystallographic information for hydroxypropyl-L-gluconamide (**1**) form I.

Table S4: Crystallographic information for hydroxypropyl-D-gluconamide (**1**).

Table S5: Crystallographic information for hydroxypropyl-L-gluconamide (**1**) form II.

Table S6: Crystallographic information for 3-hydroxypropylammonium hydrogen sulfate.

Table S7: Crystallographic information for 3-hydroxypropylammonium sulfonate.

Table S8: Crystallographic information for 3-hydroxypropylammonium hydrogen oxalate.

Table S9: Crystallographic information for guanidine carbonate methanol solvate.

Table S10: Crystallographic information for *N,N'*-ethylene bis-L-gluconamide.

Table S11: Crystallographic information for L-cysteic acid dimethylsulfoxide solvate.

Table S1: The potential excess enthalpy of mixing of the 3 components of the haircare solution with all the amino acids in hair calculated using COSMOquick.[1]

Hydroxypropyl-L-gluconamide		L-gluconic acid		3-Amino-1-propanol	
Co-former	ΔH_{mix}	Co-former	ΔH_{mix}	Co-former	ΔH_{mix}
L-lysine	-3.088	*L*-lysine	-3.222	*L*-aspartic acid	-2.559
L-arginine	-1.378	*L*-arginine	-1.951	*L*-tyrosine	-1.353
L-threonine	-0.703	*L*-histidine	-1.249	*L*-glutamic acid	-1.321
L-cysteine	-0.617	*L*-threonine	-1.109	*L*-cysteic acid	-1.121
L-histidine	-0.572	*L*-proline	-1.107	*L*-serine	-0.999
L-proline	-0.330	*L*-cysteine	-0.872	*L*-cysteine	-0.528
L-aspartic acid	-0.286	*L*-alanine	-0.753	*L*-threonine	-0.318
L-valine	-0.053	*L*-valine	-0.749	*L*-histidine	-0.216
L-isoleucine	-0.019	*L*-isoleucine	-0.714	*L*-leucine	-0.196
L-alanine	-0.012	glycine	-0.671	*L*-methionine	-0.175
L-leucine	0.042	*L*-leucine	-0.629	*L*-valine	-0.164
L-serine	0.068	*L*-methionine	-0.523	*L*-phenylalanine	-0.149
glycine	0.070	*L*-phenylalanine	-0.495	*L*-isoleucine	-0.148
L-tyrosine	0.146	*L*-tyrosine	-0.206	*L*-arginine	-0.086
L-glutamic acid	0.199	*L*-glutamic acid	-0.158	*L*-alanine	-0.083
L-phenylalanine	0.231	*L*-serine	-0.110	*L*-proline	-0.076
L-methionine	0.232	*L*-aspartic acid	0.005	glycine	-0.040
L-cysteic acid	0.525	*L*-cysteic acid	0.284	*L*-lysine	0.065

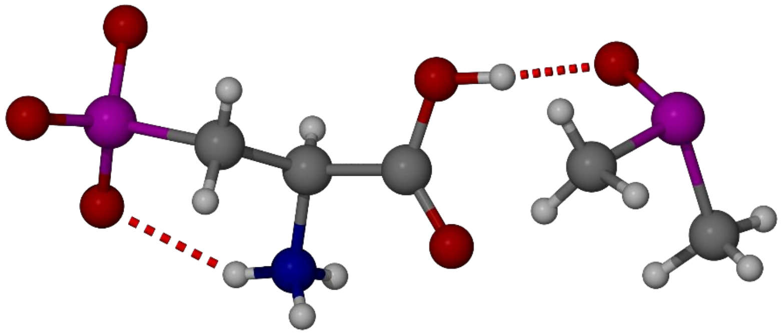

Figure S1: The X-ray crystal structure of a DMSO solvate of *L*-cysteic acid.

Scheme S1: Aniline, benzyl alcohol and the aniline and amine derivatives used for the second gel screen.

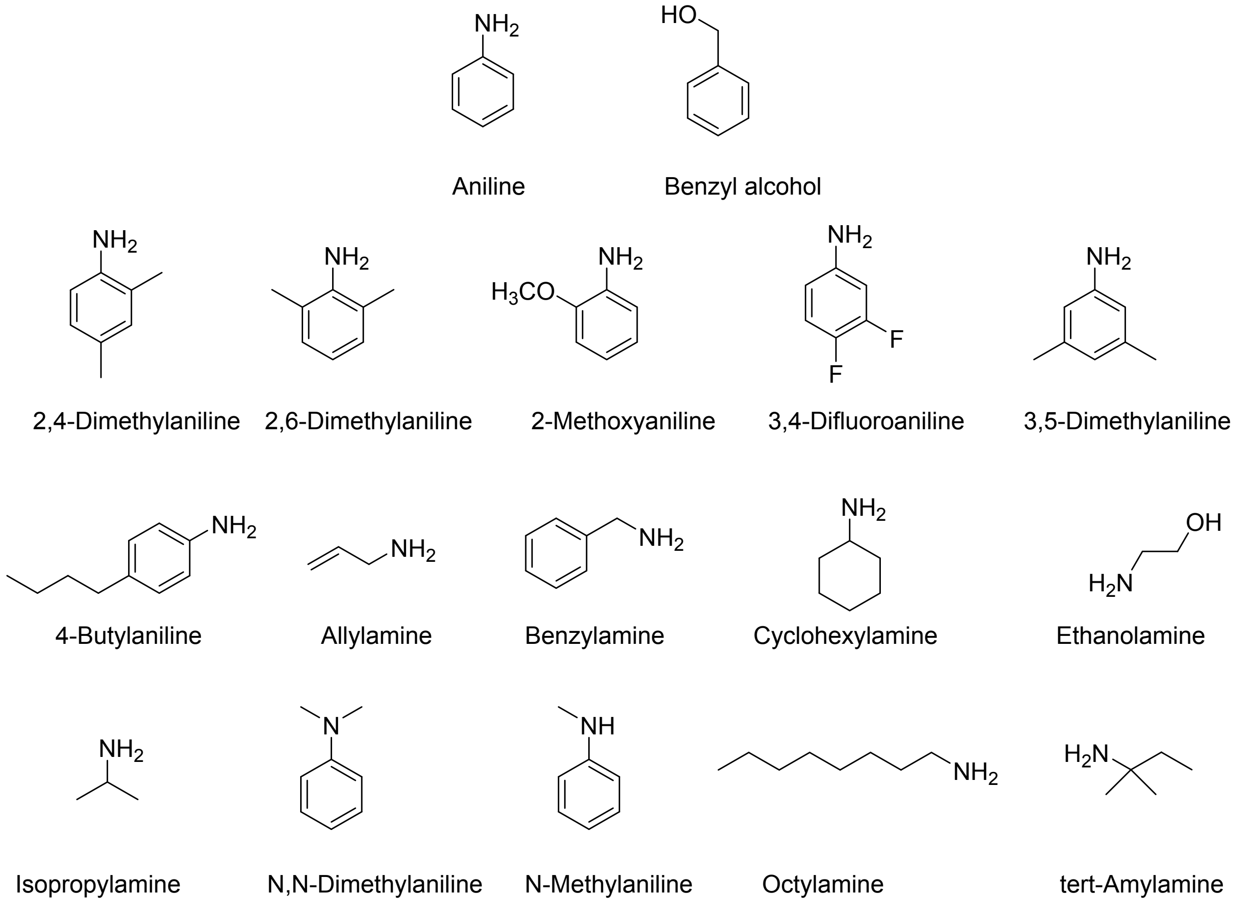

Table S2: Gel screen of **1** with a range of aniline and amine derivatives with varying wt%. G = gel, PG = partial gel, S = solution, ND = not dissolved, P = Precipitate, VL = viscous liquid.

Solvent	Result	wt%	Solvent	Result	wt%
2,4-Dimethylaniline	G	2.5	Cyclohexylamine	P	19
2,6-Dimethylaniline	PG	2.5	Ethanolamine	VL	5
2-Methoxyaniline	PG	2.5	Ethylene diamine	S	2
3,4-Difluoroaniline	G	2	Isopropylamine	L	22
3,5-Dimethylaniline	PG	15	*N,N*-Dimethylaniline	P	5
4-Butylaniline	G	2.5	*N*-Methylaniline	PG	17
Allylamine	S	2	Octylamine	P	15
Benzylamine	P	17	*Tert*-amylamine	S	2

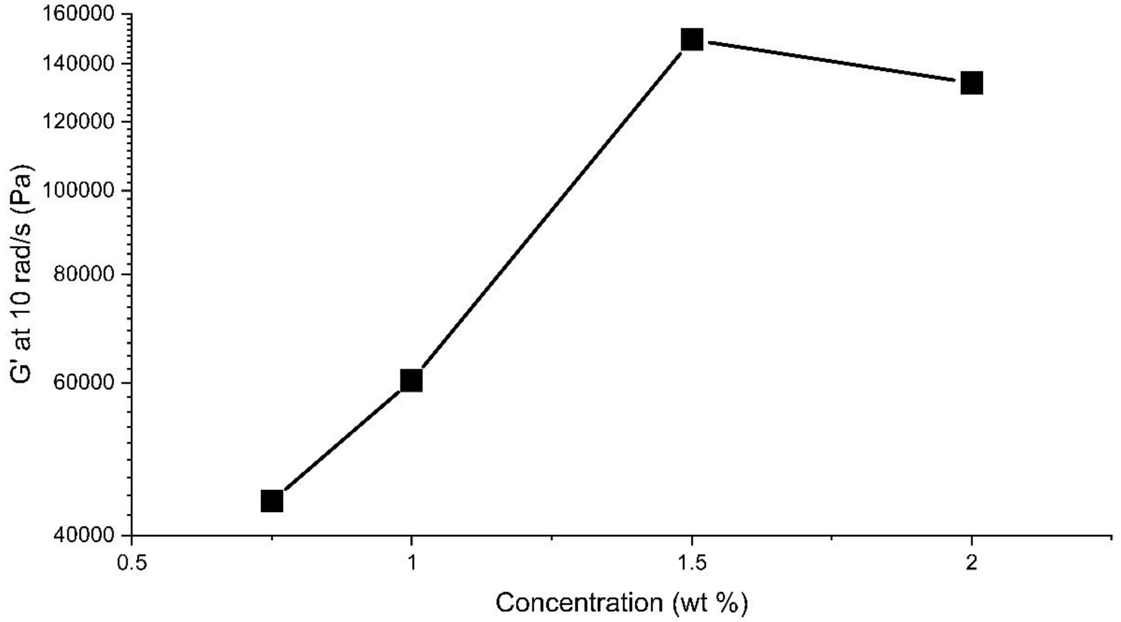

Figure S2: The variation of G' at 10 rad/s from the oscillatory frequency sweep for different concentrations of compound **1** in aniline.

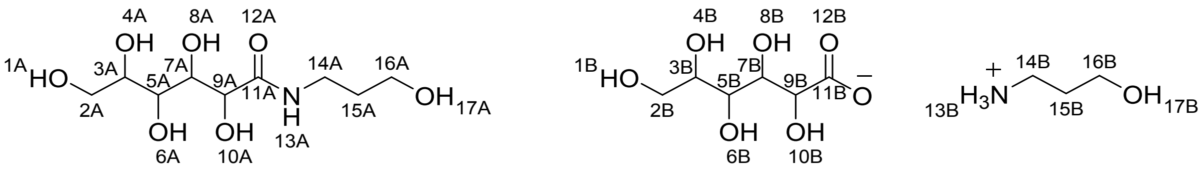

Figure S3: The labelled diagram of **1** and the gluconate salt for NMR assignment.

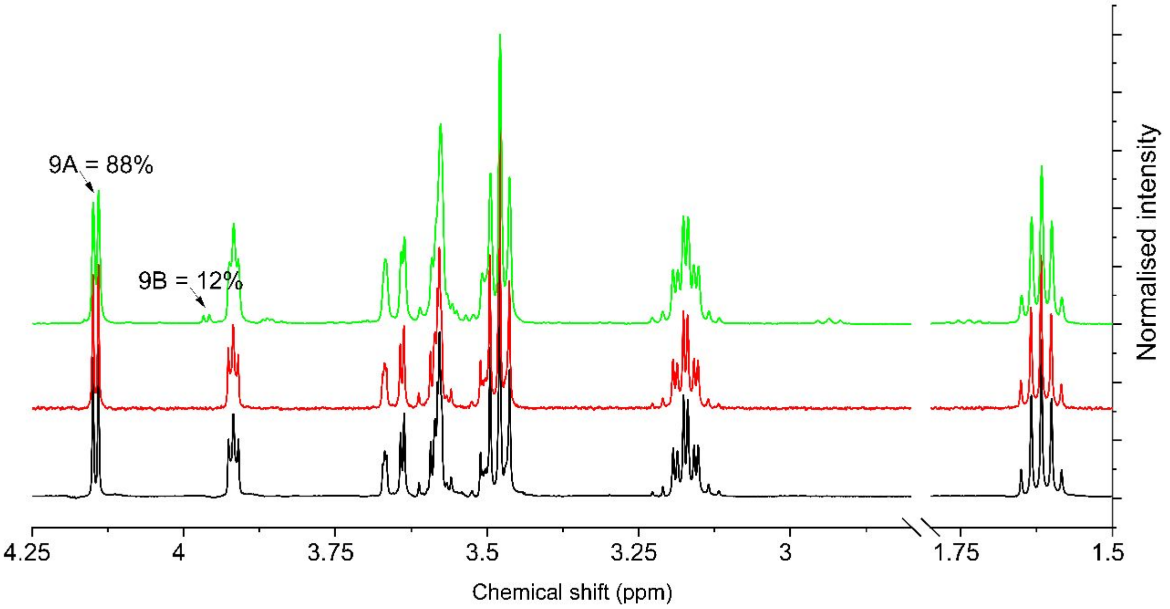

Figure S4: The ^{1}H NMR spectrum of **1** (black), **1** aniline xerogel (red) and **1** benzyl alcohol xerogel (green). Two peaks are assigned based on the labelling in **Figure** to show the ratio of **1** to the salt decomposition product.

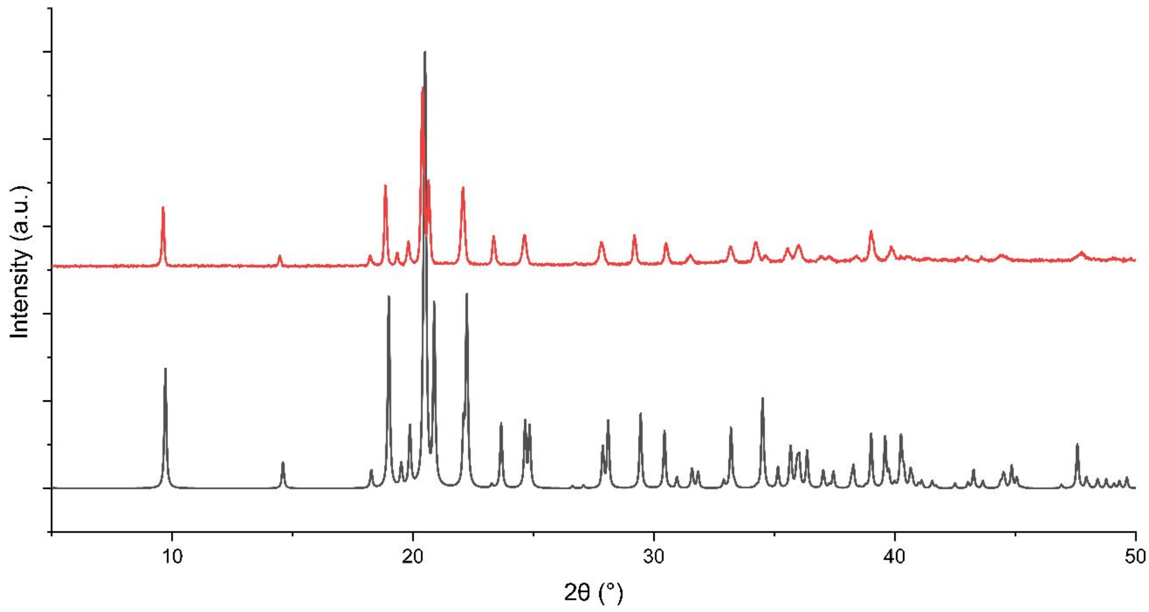

Figure S5: XRPD patterns of the *N,N'*-ethylene bis-L-gluconamide with the pattern calculated from the single crystal structure at 120 K (black) and the bulk powder at ambient temperature (red).

Table S3: Crystallographic information for hydroxypropyl-*L*-gluconamide (**1**) form I.

Crystal Information	
Empirical formula	$C_9H_{19}NO_7$
Formula weight	253.25
Temperature/K	120.0
Crystal system	monoclinic
Space group	$P2_1$
a/Å	4.6468(2)
b/Å	13.9198(7)
c/Å	8.9183(5)
α/°	90
β/°	101.1403(19)
γ/°	90
Volume/Å^3	565.99(5)
Z	2
ρ_{calc}g/cm^3	1.486
μ/mm^{-1}	0.128
F(000)	272.0
Crystal size/mm^3	0.28 × 0.18 × 0.11
Radiation	Mo Kα (λ = 0.71073)
2Θ range for data collection/°	4.656 to 59.998
Index ranges	$-6 \leq h \leq 6$, $-19 \leq k \leq 19$, $-12 \leq l \leq 12$
Reflections collected	12795
Independent reflections	3305 [R_{int} = 0.0325, R_{sigma} = 0.0294]
Data/restraints/parameters	3305/1/230
Goodness-of-fit on F^2	1.037
Final R indexes [I>=2σ (I)]	R_1 = 0.0325, wR_2 = 0.0805
Final R indexes [all data]	R_1 = 0.0367, wR_2 = 0.0823
Largest diff. peak/hole / e Å^{-3}	0.31/-0.23
Flack parameter	-0.5(7)

Table S4: Crystallographic information for hydroxypropyl-D-gluconamide (**1**).

Crystal Information	
Empirical formula	$C_9H_{19}NO_7$
Formula weight	253.25
Temperature/K	120.0
Crystal system	monoclinic
Space group	$P2_1$
a/Å	4.64620(10)
b/Å	13.9212(4)
c/Å	8.9163(3)
α/°	90
β/°	101.1335(11)
γ/°	90
Volume/Å^3	565.86(3)
Z	2
ρ_{calc}g/cm^3	1.486
μ/mm^{-1}	0.128
F(000)	272.0
Crystal size/mm^3	0.29 × 0.1 × 0.03
Radiation	Mo Kα (λ = 0.71073)
2Θ range for data collection/°	5.852 to 60
Index ranges	$-6 \le h \le 6$, $-19 \le k \le 19$, $-12 \le l \le 12$
Reflections collected	13525
Independent reflections	3268 [R_{int} = 0.0403, R_{sigma} = 0.0332]
Data/restraints/parameters	3268/1/230
Goodness-of-fit on F^2	1.021
Final R indexes [I>=2σ (I)]	R_1 = 0.0366, wR_2 = 0.0924
Final R indexes [all data]	R_1 = 0.0375, wR_2 = 0.0934
Largest diff. peak/hole / e Å^{-3}	0.34/-0.18
Flack parameter	0.0(8)

Table S5: Crystallographic information for hydroxypropyl-*L*-gluconamide (**1**) form II.

Crystal Information	
Empirical formula	$C_9H_{19}NO_7$
Formula weight	253.25
Temperature/K	120.0
Crystal system	monoclinic
Space group	$P2_1$
a/Å	9.5157(4)
b/Å	5.0795(2)
c/Å	24.2667(10)
α/°	90
β/°	96.4629(14)
γ/°	90
Volume/Å^3	1165.48(8)
Z	4
ρ_{calc}g/cm^3	1.443
μ/mm^{-1}	0.124
F(000)	544.0
Crystal size/mm^3	0.15 × 0.05 × 0.01
Radiation	MoKα (λ = 0.71073)
2Θ range for data collection/°	4.308 to 57.99
Index ranges	$-12 \leq h \leq 12$, $-6 \leq k \leq 6$, $-33 \leq l \leq 33$
Reflections collected	19083
Independent reflections	6127 [R_{int} = 0.0501, R_{sigma} = 0.0686]
Data/restraints/parameters	6127/68/363
Goodness-of-fit on F^2	1.028
Final R indexes [I>=2σ (I)]	R_1 = 0.0514, wR_2 = 0.0951
Final R indexes [all data]	R_1 = 0.0944, wR_2 = 0.1110
Largest diff. peak/hole / e Å^{-3}	0.29/-0.23
Flack parameter	0.7(7)

Table S6: Crystallographic information for 3-hydroxypropylammonium hydrogen sulfate.

Crystal Information	
Empirical formula	$C_3H_{11}NO_5S$
Formula weight	173.19
Temperature/K	120.0
Crystal system	monoclinic
Space group	$P2_1/m$
a/Å	5.3514(3)
b/Å	6.9661(4)
c/Å	9.6220(5)
α/°	90
β/°	98.976(2)
γ/°	90
Volume/Å^3	354.30(3)
Z	2
ρ_{calc}g/cm^3	1.623
μ/mm^{-1}	0.426
F(000)	184.0
Crystal size/mm^3	0.15 × 0.08 × 0.01
Radiation	MoKα (λ = 0.71073)
2Θ range for data collection/°	4.286 to 59.982
Index ranges	$-7 \leq h \leq 7$, $-9 \leq k \leq 9$, $-13 \leq l \leq 13$
Reflections collected	6372
Independent reflections	1104 [R_{int} = 0.0377, R_{sigma} = 0.0262]
Data/restraints/parameters	1104/0/83
Goodness-of-fit on F^2	1.146
Final R indexes [I>=2σ (I)]	R_1 = 0.0306, wR_2 = 0.0693
Final R indexes [all data]	R_1 = 0.0343, wR_2 = 0.0708
Largest diff. peak/hole / e Å^{-3}	0.45/-0.41

Table S7: Crystallographic information for 3-hydroxypropylammonium sulfonate.

Crystal Information	
Empirical formula	$C_4H_{13}NO_4S$
Formula weight	171.21
Temperature/K	120.0
Crystal system	monoclinic
Space group	$P2_1$
a/Å	5.1527(2)
b/Å	21.5379(10)
c/Å	7.1287(3)
α/°	90
β/°	91.6578(19)
γ/°	90
Volume/$Å^3$	790.80(6)
Z	4
$\rho_{calc}g/cm^3$	1.438
μ/mm^{-1}	0.372
F(000)	368.0
Crystal size/mm^3	0.21 × 0.07 × 0.01
Radiation	MoKα (λ = 0.71073)
2Θ range for data collection/°	3.782 to 55.998
Index ranges	$-6 \leq h \leq 6$, $-28 \leq k \leq 28$, $-9 \leq l \leq 9$
Reflections collected	18888
Independent reflections	18888 [R_{int} = 10.40, R_{sigma} = 0.1016]
Data/restraints/parameters	18888/1/187
Goodness-of-fit on F^2	1.018
Final R indexes [I>=2σ (I)]	R_1 = 0.0665, wR_2 = 0.1600
Final R indexes [all data]	R_1 = 0.0940, wR_2 = 0.1757
Largest diff. peak/hole / e $Å^{-3}$	1.13/-0.62
Flack parameter	0.58(9)

Table S8: Crystallographic information for 3-hydroxypropylammonium hydrogen oxalate.

Crystal Information	
Empirical formula	$C_5H_{11}NO_5$
Formula weight	165.15
Temperature/K	120.0
Crystal system	monoclinic
Space group	$P2_1/n$
a/Å	5.6912(4)
b/Å	7.1078(5)
c/Å	19.2926(14)
α/°	90
β/°	90.414(3)
γ/°	90
Volume/Å^3	780.40(10)
Z	4
ρ_{calc}g/cm^3	1.406
μ/mm^{-1}	0.126
F(000)	352.0
Crystal size/mm^3	0.11 × 0.1 × 0.02
Radiation	MoKα (λ = 0.71073)
2Θ range for data collection/°	4.222 to 59.996
Index ranges	$-8 \leq h \leq 8, -9 \leq k \leq 10, -27 \leq l \leq 27$
Reflections collected	12905
Independent reflections	2255 [R_{int} = 0.0450, R_{sigma} = 0.0351]
Data/restraints/parameters	2255/0/144
Goodness-of-fit on F^2	1.061
Final R indexes [I>=2σ (I)]	R_1 = 0.0414, wR_2 = 0.0925
Final R indexes [all data]	R_1 = 0.0553, wR_2 = 0.0993
Largest diff. peak/hole / e Å^{-3}	0.42/-0.32

Table S9: Crystallographic information for guanidine carbonate methanol solvate.

Crystal Information	
Empirical formula	$C_4H_{16}N_6O_4$
Formula weight	212.23
Temperature/K	120.0
Crystal system	orthorhombic
Space group	$P2_12_12_1$
a/Å	7.1149(3)
b/Å	11.6098(4)
c/Å	13.7967(5)
α/°	90
β/°	90
γ/°	90
Volume/Å^3	1139.64(7)
Z	4
ρ_{calc}g/cm^3	1.237
μ/mm^{-1}	0.107
F(000)	456.0
Crystal size/mm^3	0.21 × 0.06 × 0.01
Radiation	MoKα (λ = 0.71073)
2Θ range for data collection/°	4.586 to 59.998
Index ranges	$-10 \leq h \leq 10$, $-16 \leq k \leq 16$, $-19 \leq l \leq 19$
Reflections collected	20652
Independent reflections	3319 [R_{int} = 0.0471, R_{sigma} = 0.0326]
Data/restraints/parameters	3319/0/180
Goodness-of-fit on F^2	1.103
Final R indexes [I>=2σ (I)]	R_1 = 0.0415, wR_2 = 0.0956
Final R indexes [all data]	R_1 = 0.0487, wR_2 = 0.0985
Largest diff. peak/hole / e Å^{-3}	0.19/-0.23
Flack parameter	0.3(6)

Table S10: Crystallographic information for *N,N'*-ethylene bis-L-gluconamide.

Crystal Information	
Empirical formula	$C_{14}H_{28}N_2O_{12}$
Formula weight	416.38
Temperature/K	120.0
Crystal system	monoclinic
Space group	C2
a/Å	9.7045(4)
b/Å	5.0273(2)
c/Å	18.1838(7)
α/°	90
β/°	90.9710(10)
γ/°	90
Volume/Å^3	887.01(6)
Z	2
ρ_{calc}g/cm^3	1.559
μ/mm^{-1}	0.137
F(000)	444.0
Crystal size/mm^3	0.21 × 0.17 × 0.12
Radiation	MoKα (λ = 0.71073)
2Θ range for data collection/°	8.658 to 59.996
Index ranges	$-12 \leq h \leq 13$, $-7 \leq k \leq 7$, $-25 \leq l \leq 25$
Reflections collected	10231
Independent reflections	2537 [R_{int} = 0.0260, R_{sigma} = 0.0223]
Data/restraints/parameters	2537/1/187
Goodness-of-fit on F^2	1.105
Final R indexes [I>=2σ (I)]	R_1 = 0.0252, wR_2 = 0.0662
Final R indexes [all data]	R_1 = 0.0256, wR_2 = 0.0665
Largest diff. peak/hole / e Å^{-3}	0.34/-0.15
Flack parameter	0.0(2)

Table S11: Crystallographic information for *L*-cysteic acid dimethylsulfoxide solvate.

Crystal Information	
Empirical formula	$C_5H_{13}NO_6S_2$
Formula weight	247.28
Temperature/K	120.0
Crystal system	monoclinic
Space group	$P2_1$
a/Å	6.5483(3)
b/Å	7.9607(3)
c/Å	9.8718(4)
α/°	90
β/°	93.5090(13)
γ/°	90
Volume/Å^3	513.64(4)
Z	2
ρ_{calc}g/cm^3	1.599
μ/mm^{-1}	0.523
F(000)	260.0
Crystal size/mm^3	0.45 × 0.34 × 0.14
Radiation	Mo Kα (λ = 0.71073)
2Θ range for data collection/°	6.234 to 59.998
Index ranges	$-9 \leq h \leq 9$, $-11 \leq k \leq 11$, $-13 \leq l \leq 13$
Reflections collected	8225
Independent reflections	2839 [R_{int} = 0.0388, R_{sigma} = 0.0419]
Data/restraints/parameters	2839/1/179
Goodness-of-fit on F^2	1.056
Final R indexes [I>=2σ (I)]	R_1 = 0.0337, wR_2 = 0.0880
Final R indexes [all data]	R_1 = 0.0337, wR_2 = 0.0881
Largest diff. peak/hole / e Å^{-3}	0.33/-0.29
Flack parameter	0.03(7)

References

1. A. Klamt, *Wires Comput. Mol. Sci.*, 2018, **8**, e1338.
